# Poly (ADP-ribose) polymerase inhibitor therapy and mechanisms of resistance in epithelial ovarian cancer

**DOI:** 10.3389/fonc.2024.1414112

**Published:** 2024-07-29

**Authors:** Sanat Kulkarni, Ketankumar Gajjar, Srinivasan Madhusudan

**Affiliations:** ^1^ Department of Medicine, Sandwell and West Birmingham NHS Trust, West Bromwich, United Kingdom; ^2^ Department of Gynaecological Oncology, Nottingham University Hospitals, Nottingham, United Kingdom; ^3^ Nottingham Biodiscovery Institute, School of Medicine, University of Nottingham, Nottingham, United Kingdom; ^4^ Department of Oncology, Nottingham University Hospitals, Nottingham, United Kingdom

**Keywords:** ovarian cancer, DNA repair, PARP, PARP inhibitors, synthetic lethality, resistance, DDR inhibitors

## Abstract

Advanced epithelial ovarian cancer is the commonest cause of gynaecological cancer deaths. First-line treatment for advanced disease includes a combination of platinum-taxane chemotherapy (post-operatively or peri-operatively) and maximal debulking surgery whenever feasible. Initial response rate to chemotherapy is high (up to 80%) but most patients will develop recurrence (approximately 70-90%) and succumb to the disease. Recently, poly-ADP-ribose polymerase (PARP) inhibition (by drugs such as Olaparib, Niraparib or Rucaparib) directed synthetic lethality approach in *BRCA* germline mutant or platinum sensitive disease has generated real hope for patients. PARP inhibitor (PARPi) maintenance therapy can prolong survival but therapeutic response is not sustained due to intrinsic or acquired secondary resistance to PARPi therapy. Reversion of BRCA1/2 mutation can lead to clinical PARPi resistance in BRCA-germline mutated ovarian cancer. However, in the more common platinum sensitive sporadic HGSOC, the clinical mechanisms of development of PARPi resistance remains to be defined. Here we provide a comprehensive review of the current status of PARPi and the mechanisms of resistance to therapy.

## 1 Introduction

The development of poly(ADP-ribose) polymerase (PARP) inhibitors has led to a paradigm shift in the management of solid tumours which harbour deficiencies within the homologous recombination repair pathway (HRD), such as those with mutations in the BRCA1/2 genes (1). By exploiting an overreliance on alternative repair pathways within these tumours, PARP inhibitors (PARPi) can specifically target cancer cells whilst minimising systemic toxicity (2). Successes in pre-clinical studies and their subsequent clinical approval have translated into improved patient outcomes for patients established on PARPi, across solid tumour types (3).

Over 300,000 women worldwide are newly diagnosed with epithelial ovarian cancer each year; of the five histopathological groups, the majority are of the high-grade serous histological subtype (HGSOC) (4). Most patients are diagnosed at an advanced stage due to the diagnostic challenge posed by its often vague and ill-defined symptoms (5). The current standard of care for advanced disease involves primary debulking surgery (where feasible) followed by adjuvant platinum-based chemotherapy with the addition of anti-angiogenic agents in some cases. Despite therapeutic advances, survival of such patients remains poor with 5-year survival rates of 36% and 17% for stage III and IV disease respectively (6). However, real-world evidence from over 2000 advanced ovarian cancer patients demonstrated that 36% had mutations in BRCA1/2 or HRD and would thus be amenable to PARPi therapy (7). The application of this precision oncology strategy has subsequently improved the outlook for patients with this devastating disease (8).

However, over 40% of patients with BRCA1/2 deficiency demonstrate intrinsic resistance to PARP inhibition, manifesting as a failure to respond to treatment (9). Moreover, the vast majority of responsive patients eventually develop acquired resistance to PARPi therapy (10). This creates significant challenges in managing this ever growing patient cohort in the clinical setting.

Consequently, this review aims to summarise the evidence, from both *ex vivo* and *in vivo* studies, relating to the mechanisms of PARPi resistance and their influences on tumour biology. The review will also discuss the management strategies for advanced ovarian cancer patients with PARPi resistance and explore emerging treatments for such tumours.

## 2 DNA damage response

### 2.1 DNA repair pathways

The success of PARP inhibitors in treating ovarian cancer lies within the DNA damage response (DDR). DNA is constantly undergoing damage from endogenous sources, such as reactive oxygen species or replication errors, and exogenous causes, including chemotherapeutic agents (11). Broadly, there are six major DNA repair pathways, each used according to the type of DNA lesion sustained to maintain genomic integrity. Although relevant pathways are briefly outlined below, detailed discussion of each of these processes can be found in (12).

#### 2.1.1 Base excision repair

The majority of DNA lesions occur within one of the two DNA strands, usually as a result of single-strand breaks (SSBs) in the phosphate backbone or modification of a nitrogenous base (13). Such damage is, in most cases, repaired by the base excision repair (BER) or nucleotide excision repair (NER) pathways. BER proceeds as follows: a specific DNA glycosylase excises the base at the damaged site where AP-endonuclease 1 (APE1) then incises the phosphodiester backbone. The deoxy-ribose phosphate remnant is subsequently removed by DNA polymerase-β (polβ) (short-patch repair) or flap-endonuclease 1 (FEN1) (long-patch repair) before the correct base is inserted by DNA polymerases and the strand is resealed by DNA ligases (14, 15).

PARP1 plays a vital role within SSB repair, often considered a sub-pathway of BER. SSBs can result from reactive oxygen species or replication-associated damage; SSBs arising as intermediates from BER do not require PARP1 for repair (13, 16). PARP1 is responsible for the initial detection of the SSB, binding to the free 5’-end using its zinc finger domain. After binding, PARP catalyses the addition of poly(ADP-ribose) to itself (autoPARylation) and other effector proteins whilst also recruiting XRCC1. PARP1 subsequently rapidly dissociates from the site of damage due to charge repulsion (17). XRCC1 then acts as a scaffold for the remaining enzymes. Finally, gap filling and ligation occur as in the BER pathway (13).

#### 2.1.2 Nucleotide excision repair

In contrast to BER, nucleotide excision repair (NER) corrects lesions which cause major distortions in the helical structure of DNA (18). First the distorting damage is detected by a variety of sensor proteins, such as XPC, which recruits the transcription factor IIH (TFIIH). TFIIH utilises its helicases to unwind DNA around the lesion before dual incisions are made bilaterally by endonucleases. The resultant oligomer is removed and gap filling and ligation are performed by polymerases and ligases respectively (19–21).

#### 2.1.3 Mismatch repair

Whilst DNA polymerases possess intrinsic proofreading activity, replication-associated errors such as mismatched base pairs and insertion-deletion loops may remain unrepaired. In this circumstance, the mismatch repair (MMR) pathway is responsible for correction such lesions through the actions of the MSH2-MSH6/MSH2-MSH3 and MLH1-PMS2 heterodimer complexes (22–25). Germline mutations in MMR genes cause Lynch syndrome, in which patients carry an 8% lifetime risk of ovarian cancer compared to just 1.4% in the unaffected population (26).

#### 2.1.4 Non-homologous end joining

Although less common than SSBs, double strand breaks (DSBs) in DNA carry greater risks to the overall maintenance of genomic integrity (11). DSBs can result from ionising radiation, chemotherapeutics or in physiological circumstances such as V(D)J recombination. Broadly, there are two major repair pathways for DSBs in humans: non-homologous end joining (NHEJ) and homologous recombination (HR). The former is used more commonly (80% of the time), the exception being at DNA replication forks and in complex breaks where HR is preferred (27–29).

NHEJ proceeds as follows. The initial DSB is sensed by the Ku70/80 heterodimer which rapidly binds and recruits numerous downstream NHEJ repair factors, including DNA-dependent protein kinase catalytic subunit (DNA-PKcs) to form a DNA-PK holoenzyme and synaptic complex between the broken ends (30). If required, further processing of the broken ends is conducted by damage correction enzymes such as polynucleotide kinase 3’-phosphate (PNKP) (31), polymerases, and endonucleases such as Artemis (32). Similarly, in more complex breaks, gap filling by DNA polymerases λ and μ may be necessary prior to ligation; this is performed after binding to Ku at their C-terminus BRCA1 domains (BRCT) (33). Finally, rejoining of the broken ends is performed by DNA ligase IV, stabilised and stimulated by XRCC4 (34, 35). Notably, NHEJ can occur in either a template-dependent or independent manner with the latter carrying a greater risk of error (36). However, the flexibility of NHEJ pathway factors mean the process is likely more accurate than previously suspected (37).

#### 2.1.5 Homologous recombination

Homologous recombination (HR) repairs DSBs with higher fidelity than NHEJ as its use of a homologous template strand ensures high accuracy. HR repair proceeds through the following steps. Initial recognition of the DSB and resection from 5’ to 3’ on one strand of the DSB ends is conducted by the Mre11-Rad50-Xrs2 complex resulting in single-stranded DNA (ssDNA) tails (38). Replication Protein A (RPA) then coats ssDNA before being replaced by Rad51 in a BRCA2-dependent manner. Rad51 subsequently forms helical filaments on ssDNA to act as a nucleoprotein scaffold (38). The ssDNA then searches for and invades the homologous sequence on the sister chromatid enabling repair synthesis using the template strand by pol η. Finally, the repaired strand dissociates from its template before being ligated to seal the DSB (12). The final stages may occur through a variety of sub-pathways including synthesis-dependent strand annealing (SDSA) or creation of Holliday junctions, both of which are reviewed elsewhere (12, 39).

#### 2.1.6 Interstrand cross-link repair

Interstrand cross-links (ICLs), in which the two DNA strands become covalently bonded, may result from treatment with platinum agents, a commonly used chemotherapy agent in ovarian cancer. Such DNA damage is repaired through a pathway known as ICL repair. In quiescent cells, ICLs are generally repaired using NER machinery and translesional synthesis by DNA polymerases. Whereas in dividing cells, ICL repair is much more complex, requiring components of the Fanconi Anaemia (FA) pathway and with significant crossover with HR. This is reviewed in more detail in (40) and (41).

### 2.2 DNA repair and cancer

The relationship between DNA repair and cancer is a double-edged sword which must remain intricately balanced. On one hand, impairments within the above pathways result in unrepaired DNA lesions, subsequently leading to DNA mutations, genomic instability and carcinogenesis (42). DNA repair deficiencies may therefore manifest through hereditary cancer syndromes (where germline-mutated) (43), such as in Lynch syndrome, or more aggressive tumours carrying a worse prognosis (following acquired mutations) (44–46).

On the other hand, up-regulation of DNA repair pathways may contribute to chemotherapeutic and radiotherapeutic resistance. These agents act through initiating DNA damage with the ultimate aim of inducing tumour cell apoptosis; promotion of DNA repair within tumours may therefore limit their effectiveness (45). For instance, upregulation of ICL repair may contribute to acquired platinum resistance in ovarian tumours (47).

Nonetheless, whilst ovarian cancers often harbour mutations in DNA repair pathways, they are often initially sensitive to platinum-based combination chemotherapy. Over time and following multiple recurrences, the majority of patients develop acquired platinum resistance leaving few further treatment options (48). Evidence from clinical trial data suggests the median time to radiologic progression following surgery and chemotherapy may be as short as 12-18 months (49). Moreover, conventional chemotherapy carries a risk of systemic toxicity and, to a lesser extent, hypersensitivity reactions (8). Although second-line agents such as liposomal doxorubicin, gemcitabine, or topotecan may offer modest improvements in outcomes, the development of platinum resistance confers a guarded prognosis.

### 2.3 Synthetic lethality

**Figure 1:**
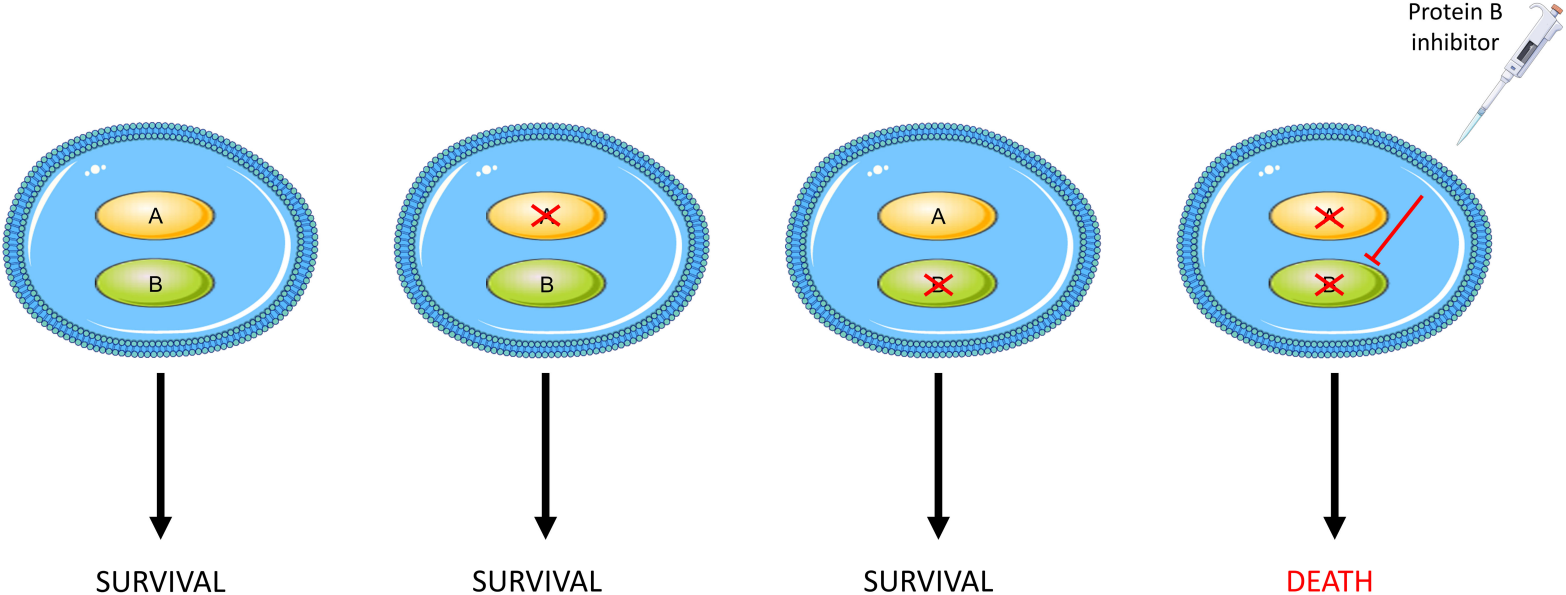
An outline of synthetic lethality. Loss of function of either gene A or B has no effect on cell survival whilst loss of function of both, either through mutations or pharmacological inhibition, results in cellular death.

Synthetic lethality, on the other hand, offers a precision strategy to improve ovarian cancer outcomes by exploiting the relationship between tumours and their DNA repair pathways. This is the situation in which cells can tolerate the loss of either one of two particular genes, and this may even confer a survival advantage, but loss of both genes results in cell death. As the aforementioned DNA repair pathways are often mutated within tumours (42), there is an overreliance on alternative, functional pathways. By selectively inhibiting such a pathway, tumour cells will continually accrue unrepaired DNA damage resulting in extensive loss of genome integrity and therefore apoptosis. In contrast, the presence of an intact pathway in normal cells prevents cell death thus ensuring selective killing of cancer cells (50). The concept of synthetic lethality is illustrated in **Figure 1**.

## 3 Biology of PARP inhibitors

The discovery of a synthetically lethal interaction between PARP1 and BRCA led to the development of PARPi, thereby demonstrating the clinical viability of this DNA-repair-directed approach for treating ovarian cancer. The role of PARP enzymes and their relationship with BRCA is discussed further in this section.

### 3.1 PARP structure and function

**Figure 2:**
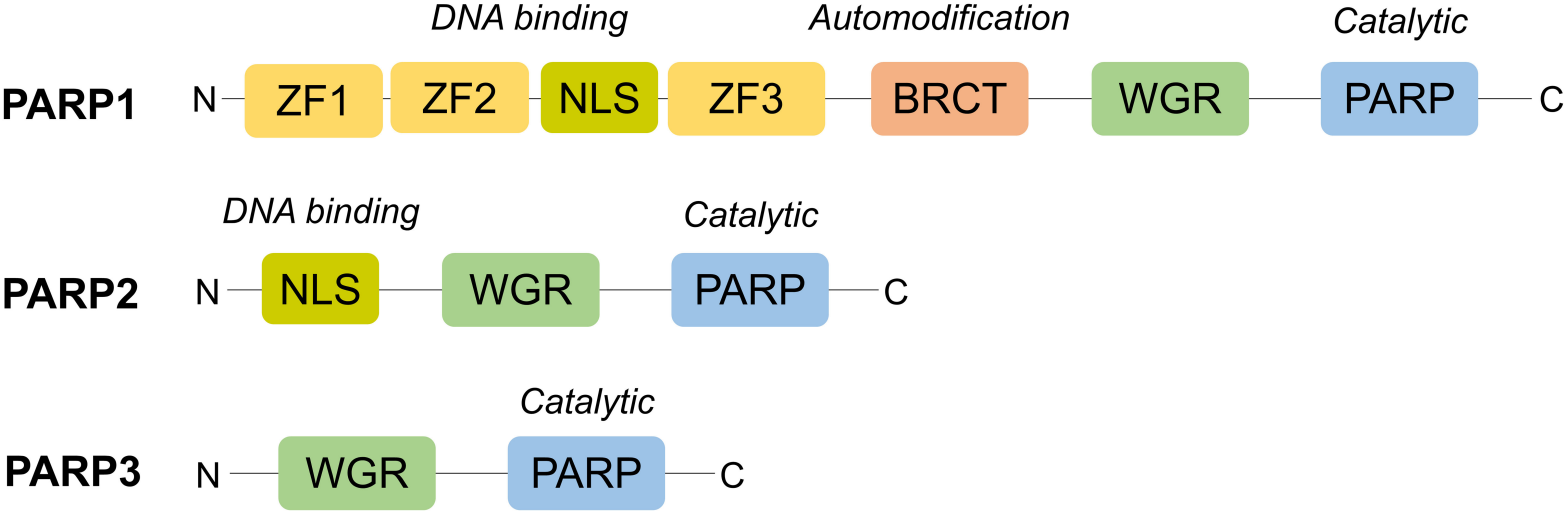
Structural domains of PARP1-3. Structurally, PARP1 is the largest (116kDa) and consists of six domains: an N-terminus with three zinc finger domains (ZF1-3) and nuclear localisation signal (NLS), an auto-modification domain with a BRCT fold, a tryptophan, glycine, arginine (WGR) motif and, at its C-terminus, a catalytic domain with its PARP signature (52). On the other hand, PARP2 and PARP3 are much smaller and possess a less complex N-terminus, lacking zinc finger domains, and no BRCT fold. However, the WGR motif is conserved across all three, highlighting its importance for their DNA-dependent activity (53).

**Figure 3:**
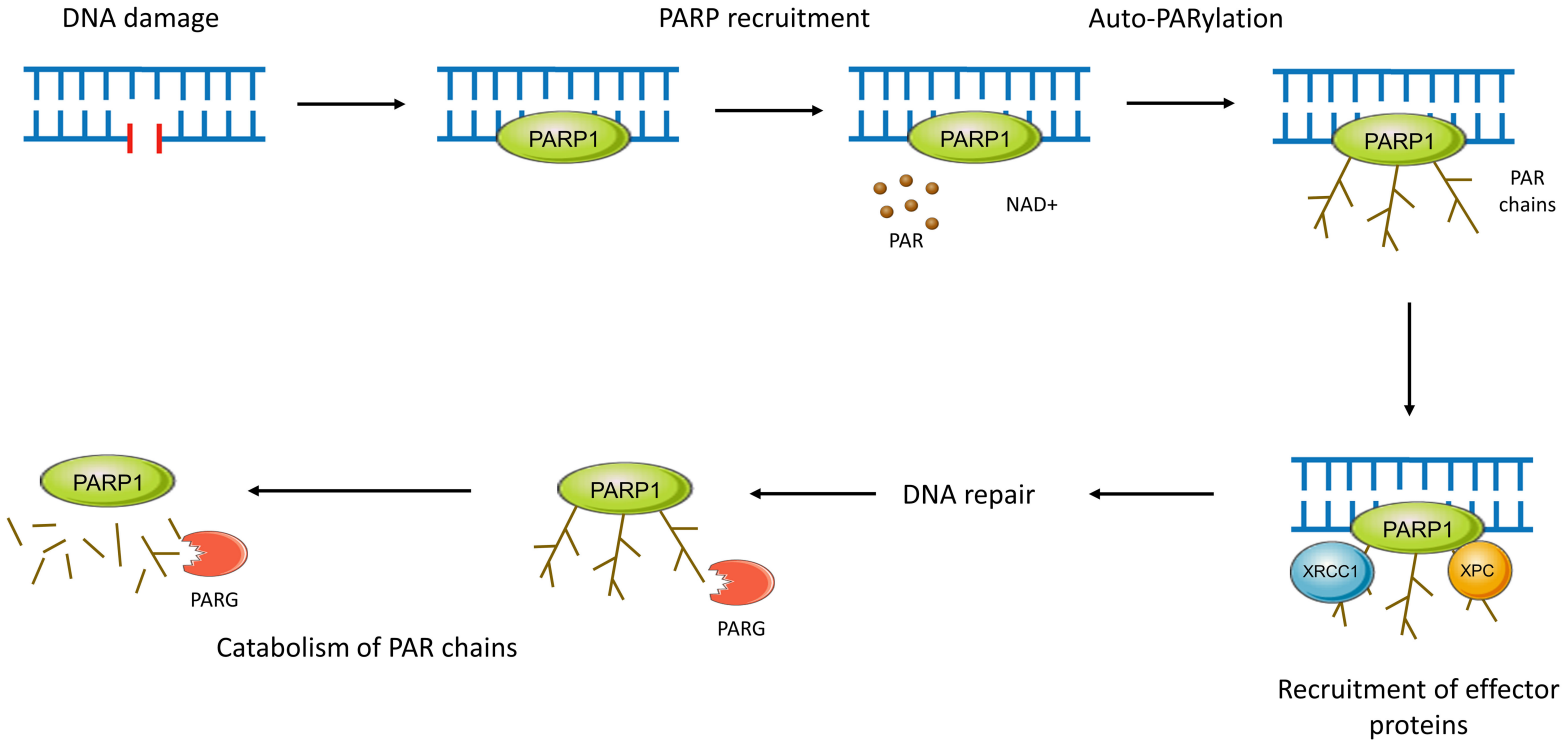
PARP activity in DNA repair. Functionally, PARPs 1-3 bind to sites of DNA damage and recruit effector proteins further downstream in the pathway. PARP1, after rapidly binding to sites of DNA damage through its WGR motif, conducts autoPARylation. This covalent attachment of around 200 repeating ADP-ribose units results in the formation of long PAR chains with branches every 20-50 units (51, 54). Recruitment of effector proteins occurs through non-covalent binding to PAR via a variety of PAR-binding motifs and domains (55). This negatively charged PAR scaffolding is then rapidly catabolised by enzymes such as PAR glycohydrolase (PARG), ensuring efficient and controlled DNA repair (51, 54, 56). PARP2 and PARP3 work in a similar manner but are preferentially recruited to DNA breaks with a 5’ phosphate where they may activate DNA ligases (57).

The PARP family of enzymes, consisting of at least 18 proteins, has diverse cellular functions (51). PARP1 through to PARP3 are DNA-dependent enzymes (**Figure 2**) and play a critical role in its repair as outlined in **Figure 3** (52–57).

### 3.2 PARP1 and DNA repair

**Figure 4:**
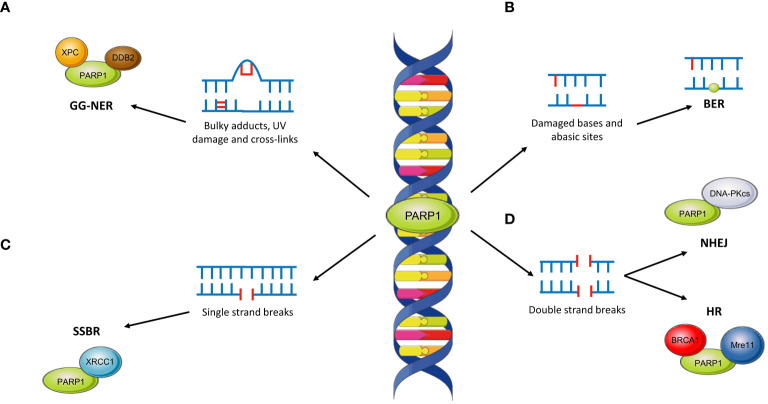
The role of PARP1 across DNA repair pathways. **(A)** PARP1 interacts with XPC and DDB2 in the repair of bulky DNA lesions such as those caused by UV damage. This is repaired through the global-genome NER pathway (GG-NER). **(B)** The role of PARP1 in BER is unclear although PARP1 may become ‘trapped’ at abasic sites, limiting the effectiveness of repair. **(C)** PARP1 plays critical roles in SSBR through interaction with XRCC1. **(D)** PARP1 is involved in both NHEJ (by interacting with DNA-PKcs) and HR (through interactions with BRCA1 and Mre11).

PARP1 has wide-ranging roles within the repair of single-strand and double-strand DNA damage across several pathways. This is reviewed in more detail in (51) but is outlined below and in **Figure 4**.

PARP has an integral role in SSBR, primarily through recruitment of XRCC1 (58). In contrast, the role of PARP within BER remains unclear. Evidence suggests that inactive ‘trapped’ PARP may limit BER pathway kinetics although there remains the possibility of downstream roles (51).

Within NER, PARP1 is responsible for damage sensing in the global genome (GG-NER) sub-pathway through interaction with XPC via PAR (59). Moreover, XPC associates with the DNA damage-binding protein 1 (DDB1)–DDB2 complex; binding of DDB2 to PARP stimulates chromatin decondensation, further facilitating repair of bulky DNA lesions (59).

Although predominantly involved in SSBR, PARP1 has further roles within DSB repair. For instance, PARP1 may facilitate recruitment of Mre11, which possesses a PAR-binding domain, to DSBs (60). Similarly, PARP1 may promote NHEJ through stimulation of DNA-PKcs (61) and recruitment of other effector proteins (62). Within HR, PARP1 may act as a controller through its interactions with a variety of key proteins. The most important of these is BRCA1; PARP1 accelerates its recruitment to DSBs although such recruitment is not always PARylation-dependent (63, 64).

### 3.3 PARP-BRCA synthetic lethality

The landmark discovery of a synthetically lethal relationship between PARP1 and BRCA was the first to be successfully exploited in the clinic, paving the way for further novel therapeutics targeting this phenomenon. PARPi such as olaparib, niraparib, rucaparib and talazoparib, suppress the activity of both PARP1 and PARP2 by competing with NAD^+^ at the enzymes’ catalytic domain (65). As a result, PARP is unable to conduct autoPARylation and dissociate from the site of damage; the ‘trapped’ PARP causes a SSB leading to replication fork collapse and therefore a DSB (66). Normal cells, with proficient HR pathways, are able to repair these DSBs. However, BRCA-mutant tumour cells lack this functionality and therefore accumulate excessive DNA damage, eventually leading to genomic instability and apoptosis. Thus, PARPi can *selectively* kill tumour cells without affecting the remaining cells in the body.

This conventional model has recently been challenged, with new evidence suggesting that PARPi synthetic lethality in BRCA-mutant cells instead stems from the accumulation of unresolved replication gaps (67).

Beyond BRCA-mutated tumours, PARPi have also been shown to demonstrate synthetic lethality in tumours with defects in other components of the HR pathway. Such HR-deficient (HRD) tumours have mutations in key HR proteins including Ataxia-telangiectasia mutated (ATM), ataxia telangiectasia and Rad3-related protein (ATR), CHK1 and CHK2 (68). The lack of functional HR means such tumours are phenotypically identical to those with BRCA mutations and thus susceptible to treatment with PARPi through the same mechanism as above.

## 4 FDA approved PARPi solid tumours

### 4.1 Pivotal clinical trials of PARPi

The pre-clinical success observed with PARPi therapy (69, 70) has translated into improved patient outcomes for ovarian cancer as evidenced through several large, phase III randomised controlled trials. Their FDA-approved indications and pivotal phase III trials are outlined in **Tables 1** and **2** respectively and discussed in detail below.

**Table 1:** FDA-approved PARP inhibitors for ovarian cancer, their approved indications and pivotal phase III, randomised trials leading to their approval.

PARP Inhibitor	Specific indication	Date of FDA approval	Pivotal phase III randomised trials for this indication
**Olaparib**	First-line maintenance for BRCAm or HRD tumours	December 2018	SOLO1
	First-line maintenance for BRCAm or HRD tumours (with bevacizumab)	May 2020	PAOLA1
	Recurrent maintenance therapy (regardless of BRCA/HRD status)	August 2017	SOLO2 and OReO
**Rucaparib**	Recurrent maintenance therapy (regardless of BRCA/HRD status)	April 2018	ARIEL3
	Third-line treatment of BRCAm tumours	December 2016	ARIEL4
**Niraparib**	First-line maintenance therapy (regardless of BRCA/HRD status)	April 2020	PRIMA
	Recurrent maintenance therapy for BRCAm tumours	March 2017	NOVA

**Table 2:** Pivotal phase III, randomised controlled trials of PARP inhibitors, stratified by each PARPi.

Trial name	Year published (most recent)	BRCA-mutated or HRD tumours only	Setting	Comparator	Sample size	mPFS for PARPi vs. comparator (months)	HR for mPFS (95% CI)	Median OS for PARPi vs comparator	HR for median OS (95% CI)
OLAPARIB									
**SOLO1**	2023	Yes	First-line maintenance	Placebo	391	56.0 vs. 13.8	0.33 (0.25-0.43)	Not reached vs. 75.2	0.55 (0.40-0.76)
**PAOLA1**	2023	No	First-line maintenance (in combination with bevacizumab)	Placebo (plus bevacizumab)	806	All: 22.1 vs. 16.6 HRD: 28.1 vs. 16.6	All: 0.33 (0.25-0.45) HRD: 0.43 (0.28-0.66)	All: 56.5 vs. 51.6 HRD: 75.2 vs. 57.3	All: 0.92 (0.76-1.12) HRD: 0.62 (0.45-0.85)
**SOLO2**	2021	Yes	Recurrent maintenance (platinum-sensitive)	Placebo	295	19.1 vs. 5.5	0.30 (0.22-0.41)	51.7 vs. 38.8	0.74 (0.54-1.00)
**OReO**	2023	No	Recurrent maintenance (after prior PARPi)	Placebo	220	BRCAm: 4.3 vs 2.8 non-BRCAm: 5.3 vs 2.8	BRCAm: 0.57 (0.37-0.87) non-BRCAm: 0.43 (0.26-0.71)	BRCAm (at 54% maturity): 20.1 vs. 20.9	0.88 (0.52-1.53)
RUCAPARIB									
**ARIEL3**	2022	No	Recurrent maintenance (platinum-sensitive)	Placebo	564	All:10.8 vs. 5.4 BRCAm:16.6 vs. 5.4 HRD: 13.6 vs. 5.4	0.36 (0.30-0.45)	45.9 vs. 47.8	0.83 (0.58-1.19)
**ARIEL4**	2022	Yes	Third-line monotherapy for relapsed disease	Chemotherapy	349	7.4 vs. 5.7	0.67 (0.52-0.86)	19.4 vs. 25.4	1.31 (1.00-1.73)
NIRAPARIB									
**PRIMA**	2023	No	First-line maintenance	Placebo	733	All: 13.8 vs. 8.2 HRD: 21.9 vs. 10.4	All: 0.62 (0.50-0.76) HRD: 0.43 (0.31-0.59)	NA	NA
**NOVA**	2023	No	Recurrent maintenance (platinum-sensitive)	Placebo	553	gBRCA: 21.0 vs. 5.5 non-gBRCA: 9.3 vs. 3.9 HRD: 12.9 vs. 3.8	gBRCA: 0.27 (0.17-0.41) non-gBRCA: 0.45 (0.34-0.61) HRD: 0.38 (0.24-0.59)	gBRCA: 40.9 vs. 38.1 non-gBRCA: 31.0 vs. 34.8 HRD: 35.6 vs. 41.4	gBRCA: 0.85 (0.61-1.20) non-gBRCA: 1.06 (0.81-1.37) HRD: 1.29 (0.85-1.95)
VELIPARIB									
**VELIA**	2019	No	First-line combination (with chemotherapy) and first-line maintenance	Chemotherapy plus placebo, chemotherapy plus veliparib followed by placebo maintenance	1140	All: 23.5 vs. 17.3 gBRCA: 34.7 vs. 22.0 HRD: 31.9 vs. 20.5	All: 0.68 (0.56-0.83) gBRCA: 0.44 (0.28-0.68) HRD: 0.57 (0.43-0.76)	NA	NA
FUZULOPARIB									
**FZOCUS-2**	2022	No	Recurrent maintenance (platinum-sensitive)	Placebo	252	All: 12.9 vs. 5.5 gBRCA: NA non-gBRCA: NA	All: 0.25 (0.17-0.36) gBRCA: 0.14 (0.07-0.28) non-gBRCA: 0.46 (0.29-0.74)	NA	NA

NA, not applicable.

#### 4.1.1 Olaparib

Olaparib is currently FDA-approved for ovarian cancer treatment in the primary and recurrent maintenance settings. It is currently indicated as a maintenance treatment following first-line platinum-based chemotherapy in those with BRCA-mutations or HRD, either with or without combination bevacizumab. This approval was based on the results of the SOLO1 (71–73) and PAOLA1 trials (74, 75). SOLO1 demonstrated significant improvements in median progression-free survival (mPFS) with olaparib over placebo (56.0 versus 13.8 months). Furthermore, at 7 years, olaparib demonstrated a clinically meaningful improvement in overall survival (OS) over placebo and was well tolerated with few severe adverse events (73).

On the other hand, the PAOLA1 trial (74, 75) evaluated the combination of olaparib and bevacizumab as primary maintenance for advanced ovarian cancer, regardless of BRCA or HRD status. This combination sought to exploit the synergism between PARPi and antiangiogenic agents (76). Anti-angiogenic agents induce hypoxia within the tumour microenvironment which in turn activates the p130/E2F4 complex; by binding to E2F consensus sequences in HR promoter genes, the activated complex thus down-regulates key HR genes such as BRCA1/2 and RAD51 (77). The combination therapy demonstrated significant improvements in mPFS as compared to placebo plus bevacizumab but only within the BRCA-mutant and HRD cohort (mPFS 37.2 vs. 17.7 months; HR 0.33; 95% CI, 0.25–0.45). Consequently, olaparib maintenance therapy is only indicated following companion diagnostic testing for BRCA-status (Myriad CDx) or HRD (FoundationOne CDx) (78). Matured data demonstrated a significant improvement in OS at 5 years in the HRD cohort only (5-year survival 65.5% vs. 48.4%; HR 0.62, 95% CI 0.45-0.85) (79).

However, in the recurrent setting, platinum-responsive ovarian cancer patients are eligible for olaparib maintenance regardless of BRCA or HRD status, based on results from the SOLO2 (75, 80) and OReO (81) phase III randomised trials. SOLO2 included BRCA-mutated patients only and demonstrated significantly improved mPFS in favour of olaparib (19.1 vs. 5.5 months; HR 0.30; 95% CI, 0.22–0.41) with an improvement in OS (although not reaching significance) (75, 80). The recently published OReO trial demonstrated the significant benefits of maintenance olaparib rechallenge over placebo, independent of BRCA-status. A total of 220 patients with relapsed, platinum-sensitive ovarian cancer (and had received prior PARPi therapy) were randomised 2:1 to receive either 300mg olaparib maintenance or placebo. Both the BRCA-mutated and non-BRCA mutated subgroups had significant improvements in mPFS with olaparib rechallenge, which was well tolerated (81).

#### 4.1.2 Rucaparib

In contrast, rucaparib has only received FDA approval for recurrent maintenance treatment or as third-line treatment for those with BRCA mutations following the results of the ARIEL3 (82, 83) and ARIEL4 (84, 85) trials respectively. Within ARIEL3, the significant benefits of rucaparib were not only observed in the HRD and BRCA-mutated group but also in the intention-to-treat population, highlighting the benefits of PARPi therapy beyond those with known DNA repair defects (82). Nevertheless, there was no significant improvement in OS across any subgroup. The ARIEL4 trial demonstrated less encouraging results for rucaparib as a third-line treatment for BRCA-mutated advanced ovarian cancer as compared to chemotherapy (dependent on platinum-sensitivity). The study found overall survival to be better in the chemotherapy arm, resulting in its withdrawal as third-line treatment in the UK. However, these results may be affected by the high rate of crossover between arms (84, 85).

#### 4.1.3 Niraparib

Niraparib is only FDA-approved as a primary maintenance therapy or as recurrent maintenance treatment for BRCA-mutated ovarian cancers. The former approval was based on the findings of the PRIMA phase III trial of genomically unstratified, platinum-sensitive ovarian cancer patients (86, 87). The data again demonstrated the benefits of PARPi even in patients without BRCA mutations or HRD status (mPFS 13.8 vs. 8.2 months; HR 0.62; 95% CI 0.50 to 0.76), resulting in its FDA approval for use without the need for companion diagnostic testing (86, 87).

In the recurrent maintenance setting, the NOVA trial initially demonstrated positive results for mPFS as compared to placebo for relapsed ovarian cancer patients regardless of germline BRCA mutation (gBRCAm) or HRD status (88). However, mature OS data showed no evidence of an improvement with niraparib over placebo, regardless of BRCA or HRD status. However, the trial was underpowered to detect differences in OS and, as such, further data is necessary to corroborate these findings (89).

#### 4.1.4 Talazoparib

Talazoparib is approved for the treatment of breast and prostate cancers but there is limited evidence supporting its use in ovarian cancer patients. In comparison to other PARPi, talazoparib possesses far greater potency by virtue of its superior PARP trapping ability (90) although this may explain its higher risk of myelosuppression (91). Whilst there have been encouraging results in single agent (92) and combination phase I clinical trials (93), later phase trials are lacking (94).

#### 4.1.5 Veliparib

Similarly, veliparib remains under investigation as a therapeutic agent for ovarian cancer and has not, as yet, received FDA approval. The phase III, placebo-controlled VELIA trial of 1140 patients with ovarian cancer demonstrated significant improvements in mPFS when veliparib was used in combination with chemotherapy followed by maintenance monotherapy as compared to chemotherapy alone (23.5 vs. 17.3 months) (95). This approach highlights the potential benefits of PARPi-chemotherapy combinations; PARPi therapy can potentiate chemotherapy-induced damage leading to greater tumour cell death. However, as discussed further below, such combinations may be limited by their toxicity profile. Benefits were observed regardless of HRD/BRCA status and the treatment was generally well tolerated (96). However, there was no clear benefit of veliparib in the combination phase alone as compared to placebo (95).

#### 4.1.6 Fuzuloparib

Fuzuloparib is a novel PARPi developed and approved in China for gBRCAm ovarian cancer following second-line or greater chemotherapy and for recurrent maintenance therapy (97). However, it has not, as yet, received FDA approval. It has been suggested that differences in its chemical structure compared to other PARPi may contribute to better stability and reduced inter-individual variability (98). The open-label, single-arm, phase II FZOCUS-3 trial (99) included 113 patients with platinum-sensitive recurrent gBRCAm ovarian cancer treated with fuzuloparib. The results demonstrated the promising efficacy of the novel PARPi in the recurrent setting; the ORR was 69.9% (95% CI: 60.6-78.2%) and mPFS was 12.0 months (95% CI: 9.3-13.9 months).

Furthermore, the double-blind, placebo-controlled phase III FZOCUS-2 trial demonstrated fuzuloparib’s benefits in the recurrent maintenance setting for platinum-sensitive ovarian cancer. In total, 252 patients who had received at least two lines of platinum-based chemotherapy were enrolled regardless of BRCA/HRD status and randomly allocated in a 2:1 manner to either fuzuloparib or placebo. The drug significantly improved mPFS across the whole study population (12.9 vs. 5.5 months; HR 0.25; 95% CI 0.17 to 0.36), regardless of BRCA status, and was generally well tolerated.

#### 4.1.7 Pamiparib

Pamiparib is also a novel PARPi developed in China and has similarly demonstrated promising antitumour activity against ovarian cancers in phase I and II trials (100). In particular, a recent open-label phase II trial of 113 patients with recurrent ovarian cancer showed encouraging evidence of efficacy. Of the 90 patients with platinum-sensitive disease, ORR was 64.6% (95% CI: 53.3-74.9%) whilst in the 23 with platinum-resistance, the ORR was 31.6% (95% CI: 12.6-56.6%) (101).

#### 4.1.8 PARPi in combination therapies

As reviewed in greater detail in (8) and briefly outlined above, PARPi have demonstrated synergism with several other therapeutic agents. Currently within the FDA-approved setting, such combination approaches remain limited to chemotherapy and anti-angiogenic agents based on the rationale and trial data discussed above.

### 4.2 Adverse effects and toxicity profile of PARP inhibitors

Across clinical trials and from real-world data, common adverse class effects of PARPi include haematological toxicity, fatigue, and nausea (102, 103). In particular, anaemia is the most commonly encountered haematological toxicity, affecting approximately 40% of patients (102, 103). This may be the result of an on-target adverse effect relating to the role of PARP2 in erythropoiesis (104). Moreover, PARPi carry a risk of neutropenia (seen in 18-30% of patients) and thrombocytopenia (seen in 8-46% of patients) (103). Notably, niraparib appears to carry the highest risk of haematological toxicity, in particular grade 3 and 4 adverse events (102). These risks appear to be heightened when PARPi are used in combination with chemotherapy agents (105).

Nausea was commonly reported within clinical trials, affecting over 70% of patients across all PARPi. However, gastrointestinal symptoms were mild overall, rarely resulting in grade 3 or 4 toxicity (102). Similarly, fatigue is a common class effect of PARPi, affecting around one-third of patients, but again rarely leads to grade 3 or 4 toxicity (103).

Toxicity generally occurs early in the treatment course, typically within the first 4-8 weeks, with approximately 7-20% of patients discontinuing the therapy. In the longer term, PARPi may carry a small risk of secondary haematological malignancies (0.5-1.4%) such as acute myeloid leukaemia and myelodysplastic syndrome, perhaps stemming from their effects on the DNA damage response (102). The development of interstitial lung disease is another long-term complication which warrants monitoring with olaparib therapy (106).

It is worthwhile noting that observed differences in their efficacy and side effect profiles may partly relate to differential ‘trapping’ ability and potency in inhibiting PARP1 catalytic activity (107). Veliparib, on the other hand, acts through inhibition of autoPARylation, highlighting the heterogeneity in PARPi’s (108, 109). Evidence from a recent network meta-analysis further supports this; olaparib demonstrated the best overall safety profile, followed by talazoparib and rucaparib with niraparib having the worst overall safety profile (91). Moreover, despite being a targeted therapy, only olaparib had a better safety profile than conventional chemotherapy, whilst PARPi in combination with an angiogenesis inhibitor had a worse overall safety profile than any PARPi monotherapy.

## 5 Mechanisms of resistance to PARP inhibitors

### 5.1 PARPi in clinical practice

Following the encouraging results from these large-scale trials (**Table 2**), PARPi are now standard practice in the management of ovarian cancer. PARPi have been shown to have significant benefits in the real-world setting over active surveillance (110, 111).

Despite these benefits, trial and real-world data show that most patients have progressive disease despite PARPi therapy. Across the discussed phase III trials, whilst most found significant benefits in PFS, many failed to show improvements in OS. This finding is likely explained by the development of resistance following prolonged PARPi therapy with real-word evidence suggesting a median time to progression between 10 and 16 months (112, 113). Evidence suggests approximately 40% of patients fail to respond to PARPi due to intrinsic resistance whilst development of acquired resistance is almost ubiquitous with prolonged therapy (9, 10). Developing our understanding of the mechanisms of PARPi resistance is critical in formulating appropriate treatment strategies for this ever-growing patient cohort.

### 5.2 Mechanisms of PARPi resistance

**Figure 5:**
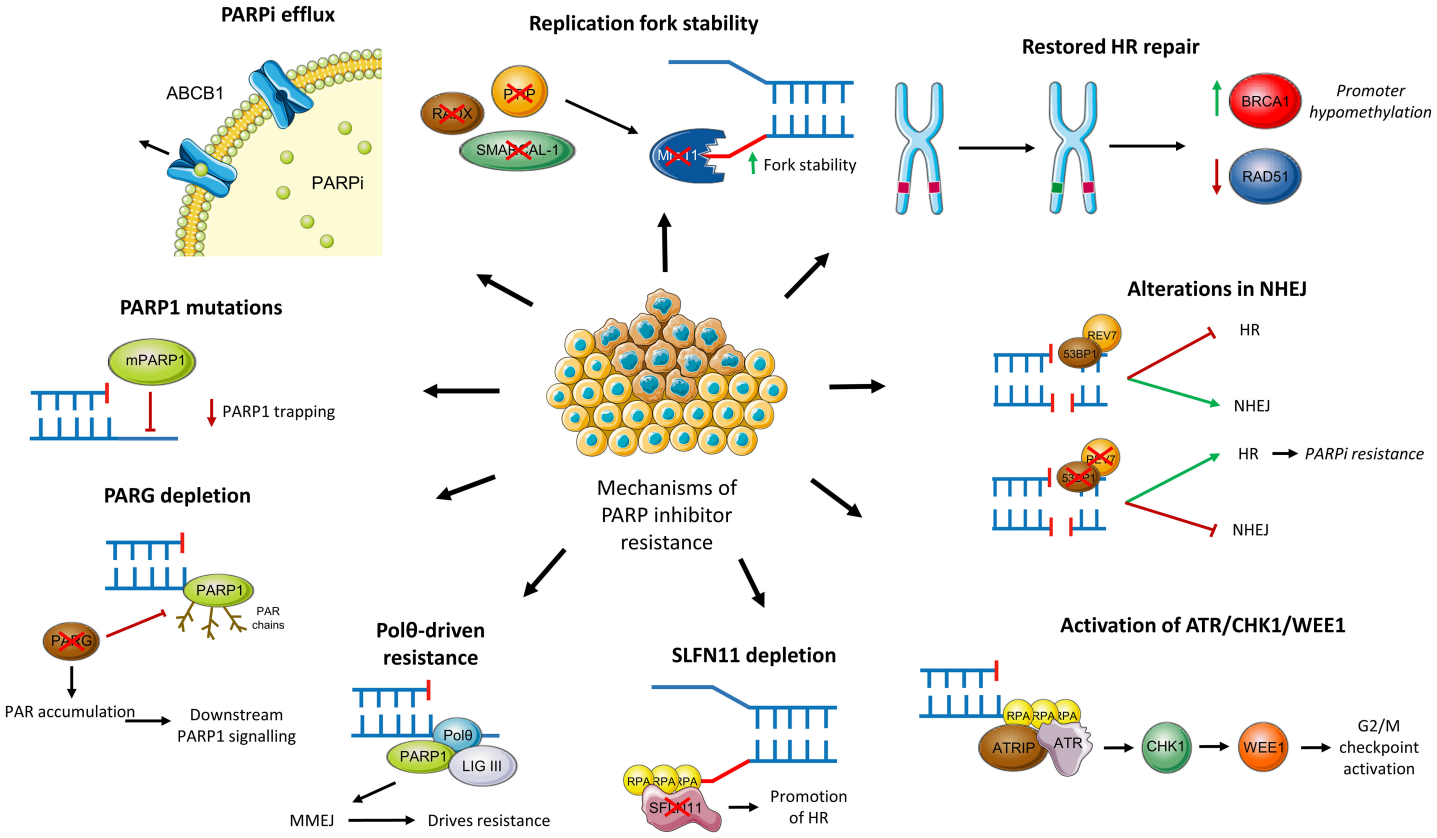
Mechanisms of PARPi resistance.

Evidence from both *in vitro* and *in vivo* studies have highlighted several potential mechanisms through which tumour cells may acquire resistance to PARPi. This section discusses these specific mechanisms of resistance to PARPi, including restored HR functionality, activation of other DNA repair pathways, alterations in the DNA damage response and mutations or depletion of PARP and PARG. These mechanisms are also summarised in **Figure 5**.

#### 5.2.1 Restoration of functional HR repair

Results from the EVOLVE trial demonstrated that reversion mutations in BRCA1, BRCA2 and RAD51B were the most common acquired genomic alterations amongst PARPi-resistant patients (19%) (114). Similarly, a meta-analysis of 269 patients with progressive solid tumours found that 26% had BRCA reversion mutations (115). Such mutations, including the c.6174delT frameshift mutation, restore the open reading frame (ORF) of BRCA resulting in functional HR repair of DSBs, thus preventing synthetic lethality with PARPi. *In vitro* studies have demonstrated that exposure to PARPi results in Darwinian selection for such mutant cells, the extent of which is governed by the frequency of drug administration (116).

Epigenetic changes may also be responsible for restoring BRCA, and therefore HR, functionality (117). In particular, reduced methylation of the BRCA1 promoter region may lead to HR recovery and resistance as evidenced by a study with patient derived xenografts (PDX) (118). Furthermore, resistance is evident in tumours with heterozygous methylation of BRCA1 whilst homozygosity or hemizygosity is predictive of PARPi response in PDX models and tumour samples (119). However, accurate assessment of BRCA methylation zygosity in patient samples is challenging and further larger scale research is necessary to corroborate these findings.

Beyond BRCA, increased expression and secondary mutations of other HR pathway components may confer resistance to PARPi (120). RAD51 is an important example of this; as outlined above, RAD51 coats ssDNA and acts as a nucleoprotein scaffold within the HR pathway (38). Importantly, RAD51 protects nascent DNA from nuclease degradation and is thus critical in preventing the accumulation of gaps at replication forks (121). Considering the new mechanistic model suggesting PARPi’s synthetic lethality occurs due to the accumulation of such gaps, it is therefore unsurprising that higher expression of RAD51 negatively correlates with PARPi responsiveness (122–124). Increased foci of RAD51 and its association with PARPi resistance is notably independent of BRCA status (124). In a similar manner to BRCA, secondary mutations in RAD51 may restore its ORF (125) whilst hypomethylation may confer PARPi resistance (126). RAD51 may therefore be a promising biomarker for predicting PARPi resistance although currently available tests are unable to detect epigenetic changes (127).

#### 5.2.2 Alterations in NHEJ

As discussed above, NHEJ is responsible for the repair of DSBs, especially so in cells lacking a functional HR pathway (HRD or BRCA-mutated). The overreliance on this more error-prone pathway contributes to genomic instability within tumours and can be exploited through synthetic lethality (2). However, mutations within key NHEJ effector proteins may lead to a reactivation of HR and thus acquired resistance to PARPi (117).

Key examples of this include TP53-binding protein (53BP1) and REV7. 53BP1 acts to inhibit HR by binding to terminal DNA and preventing excision. Normally, 53BP1 is removed by BRCA1 to facilitate HR repair. REV7 acts in a similar manner, inhibiting HR and promoting NHEJ. Consequently, loss or reduced levels of 53BP1 or REV7 promote HR repair and therefore confer PARPi resistance (128, 129). This has been demonstrated in ovarian cancer cells lines with BRCA1 mutations (130) although 53BP1 does not mediate resistance in BRCA2 mutated cells (131). Furthermore, REV7 acts as part of the shieldin complex, consisting of itself, SHLD1, SHLD2 and SHLD3. Similarly, loss of SHLD1 or SHLD2 can result in acquired resistance, highlighting the complex interplay and variety of potential mutations leading to the same overarching mechanism of resistance (132, 133). Overall, the combination of 53BP1 and BRCA status may act as a useful biomarker for PARPi resistance, although consideration must be given to the frequency and impact of downstream mutations such as within the shieldin complex (134).

In a similar manner, it has been shown in ovarian cancers that amplifications within the *CCNE1* gene (encoding cyclin E1) demonstrate poor responsiveness to PARPi. This genomic alteration was observed in 16% of resistant patients within a recent clinical trial (114). The mechanism likely stems from the mutual exclusivity of *CCNE1* amplification and HRD, enabling cells to repair DSBs (135).

#### 5.2.3 Increased activation of the ATR/CHK1/WEE1 pathway

The ataxia telangiectasia and Rad3-related (ATR)-checkpoint kinase 1 (CHK1) pathway plays vital roles within the DNA damage response. Following end resection of DSBs and at stalled replication forks, ssDNA-bound RPA recruits ATR and ATR interacting protein (ATRIP) which in turn activates CHK1. CHK1 then inhibits CDK2 during S-phase through degradation of CDC25A. This results in activation of the intra-S and G2/M phase cell-cycle checkpoints, allowing initiation of DSB repair (136). WEE1 additionally plays a role at the G2/M checkpoint by inhibiting CDK1/cyclin complexes through phosphorylation, thus preventing progression to mitosis (137). PARP inhibition results in cellular replication stress and therefore activation of ATR/CHK1 and WEE1; resistant cells rely heavily on this signalling for survival (138). This is further evidenced by ATR inhibition re-sensitising these resistant cell lines (139).

#### 5.2.4 Down-regulation of SLFN11

As confirmed in patients in the EVOLVE trial, down-regulation of Schlafen 11 (SFLN11) was observed in 7% of PARPi resistant patients (114). SFLN11 is a DNA/RNA helicase which acts at stressed replication forks to trigger replication blocks and cell death (140). Down-regulation of this protein therefore enables efficient DNA repair and therefore cell survival, even in the presence of PARPi. This has been validated through several pre-clinical studies across tumour types (141–145), demonstrating its potential as a predictive biomarker (140). Early results from a recent biomarker-selected trial for patients with SLFN11-positive end-stage small-cell lung cancer have shown the benefits of PARPi combination therapy in this particular cohort and the feasibility of SLFN11 stratification (146). However, it is unclear the extent to which this may be applied to ovarian cancer patients, particularly those with acquired PARPi resistance.

#### 5.2.5 Polθ-driven resistance

Polθ (POLQ) is responsible for conducting theta-mediated end-joining (TMEJ), also known as microhomology mediated end joining (MMEJ), which is an uncommonly used and error-prone process by which cells can repair DSBs (147). In HRD tumours, and particularly those with deficiencies in NHEJ as described above, repair of DSBs is heavily reliant on this pathway. This in turn contributes to PARPi resistance. Evidence from a cell line study of PARPi-resistant cells due to 53BP1/Shieldin defects supports this; pharmacological inhibition of polθ overcame this resistance and resulted in synthetic lethality in these cells (147).

#### 5.2.6 PARG depletion or mutations

As previously described, PARG is responsible for reversing PARylation and, as do PARPi themselves, acts to prevent the accumulation of PAR at sites of DNA damage. Loss of PARG has been shown to reduce PARP trapping, restore PAR accumulation and subsequent downstream signalling of PARP1 (148). PARG-depleted cells demonstrate an overreliance on the ATR/CHK1/WEE1 damage response pathway, highlighting a potential therapeutic target for such tumours (149, 150).

#### 5.2.7 PARP1 mutations

Point mutations in PARP1 have been shown to lead to resistance in *in vitro* studies. For instance, mutations in the zinc finger domain of PARP1 reduces binding to sites of DNA damage and therefore cytotoxic trapping (128). Moreover, point mutations outside of the zinc finger domain are though to contribute to PARPi resistance. An ovarian cancer patient with *de novo* resistance to olaparib was found to harbour a p.R591C mutation affecting the WGR domain of PARP1. It is thought that such a mutation will limit inter-domain communication within PARP1, allowing DNA binding but limiting trapping. This therefore provides some degree of clinical validation of this resistance mechanism, although widespread screening for point mutations is inherently challenging (151).

#### 5.2.8 PARPi efflux

Increased expression of the drug transporter ABCB1 (also known as MDR1) has been shown to increase efflux of PARPis and thus contribute to resistance. ABCB1 upregulation was demonstrated in 15% of PARPi-resistant patients in the EVOLVE trial (114). ABCB1 is part of the wider ATP-binding cassette (ABC) transporter superfamily and its wide range of potential substrates mean it is responsible for the efflux of many chemotherapeutic drugs (152). It is unsurprising therefore, that high expression of the transporter has been reported in drug-resistant breast and ovarian cancer (153). Within ovarian cancer cell lines, resistance to olaparib and rucaparib was positively correlated with ABCB1 expression although there was no relation with veliparib exposure (154). It has been postulated that exposure to chemotherapeutics, such as taxane agents, may upregulate ABCB1 and thus contribute to later development of PARPi resistance (155). Use of alternative PARPi which are not ABCB1 substrates, such as pamiparib, may offer a means of overcoming this resistance mechanism (156). Alternatively, use of ABCB1/MDR1 inhibitors can offer another means although pre-clinical evidence suggests this compound lacked synergy with PARPi (157).

#### 5.2.9 Restored replication fork stability

As discussed above, PARPi’s toxicity may stem from lagging strand gaps and defective processing of Okazaki fragments at replication forks (67). DNA damage impedes the replication process resulting in replication stress and slowing, or stalling, of the replication fork. Both PARP and BRCA are vital in protecting and stabilising the fork during replication stress. The latter prevents Mre11-mediated degradation of nascent DNA and therefore maintains genomic integrity. Conversely, PARP inhibition results in acceleration of the replication fork and the resultant gaps due to exhaustion of RPA pools are likely responsible for cellular toxicity (67, 128). Numerous pre-clinical studies have demonstrated that loss of key proteins, such as PTIP, SMARCAL-1 and RADX, can lead to replication fork stabilisation and therefore PARPi resistance (158–160). For instance, PTIP deficiency inhibits the recruitment of Mre11 to stalled forks, thereby protecting degradation of nascent DNA (158). Furthermore, reduced expression of these key genes has been associated with inferior outcomes in ovarian and pancreatic cancer, further underlining their importance (128).

#### 5.2.10 Future directions

However, evidence from a study of 26 HGSOC patient samples following PARPi therapy demonstrated that small scale mutations within DDR-related genes, including BRCA reversion mutations and mutations in RAD51, SHLD2 and 53BP1, were uncommon definitive mechanisms of resistance (161). Whilst *in vitro* validation of the alternative mechanisms described are necessary, exploring the role of larger scale genomic changes such as copy number variation and alterations at the transcriptional level are important next steps. Equally, further consideration of the importance of epigenetic changes, immune responses and the tumour microenvironment are necessary. Evidence from the EVOLVE study suggests that multiple resistance mechanisms may co-exist and act in parallel to confer PARPi resistance; ascertaining the extent to which each contributes (if at all) poses an additional challenge (114).

## 6 Management of PARP inhibitor resistance in the clinic

At present, there is a dearth of evidence regarding the best approach to managing patients with intrinsic or acquired PARPi resistance. There is a pressing need, therefore, for clinical trials in the post-PARPi setting. Moreover, such trials must be rationalised by pre-clinical studies which can identify actionable alterations in tumour biology following the development of PARPi resistance.

### 6.1 Current treatment approaches for PARPi resistant patients

#### 6.1.1 Platinum sensitivity and eligibility

The current clinical approach to managing PARPi-resistant patients is governed by platinum sensitivity. In general, patients are considered to be platinum sensitive where the platinum-free interval (PFI) is greater than 6 months whilst those with a PFI greater than this are labelled as platinum resistant (162). Nevertheless, the appropriateness of this temporal cut-off point has been questioned (163, 164). Progression during platinum therapy is the sole definitive marker for platinum resistance; early relapse (PFI <6 months) merely raises the likelihood. Likewise, late relapse (PFI >6 months) increases the chances of, but importantly does not guarantee, platinum sensitivity (165). Therefore, it may be more appropriate to classify patients with relapse as either ‘platinum-eligible’ or ‘platinum-ineligible’.

#### 6.1.2 Cross-resistance between PARPi and chemotherapy agents

Moving forward, further elucidating the relationship between resistance to PARPi and resistance to platinum agents and other chemotherapeutics may help in guiding management. Platinum agents induce significant DNA damage in the form of intrastrand adducts and interstrand crosslinks (ICLs); these are repaired by a variety of pathways as described above, including NER, MMR, NHEJ, HR and ICL repair (166). It therefore follows that BRCA-deficiency or HRD is associated with greater platinum sensitivity, whilst reversion mutations confer resistance to both platinum agents and PARPi (167–169). Furthermore, increased expression of the ABCB1 transporter is associated with platinum and taxane resistance, thus highlighting the significant crossover in mechanisms of cross-resistance between PARPi and chemotherapy agents (170, 171).

These findings have been corroborated in clinical trials and real-world data. *Post-hoc* analyses of the SOLO2 trial included 147 ovarian cancer patients who progressed either on olaparib or placebo and were subsequently treated with either platinum-based or non-platinum-based chemotherapy. The findings demonstrated a significantly longer time to second progression amongst those who had received placebo than those who had progressed on olaparib (12.1 vs. 6.9 months). However, this only remained significant within the platinum-based chemotherapy group (14.3 vs. 7.0 months) in contrast to the non-platinum-based chemotherapy (8.3 vs. 6.0 months) (172). This may reflect the greater crossover in resistance mechanisms between platinum agents and PARPi compared to other agents. Real-world data further support these findings (165, 173, 174). However, one study demonstrated that patients without BRCA mutations who progressed on PARPi derived significantly greater benefit from subsequent platinum therapy than those harbouring BRCA mutations (mPFS 7.5 vs. 3.5 months) (174). This finding may be explained by the development of differing PARPi resistance mechanisms between the two groups with varying degrees of cross-resistance to platinum agents. Intriguingly, 13 patients with platinum-resistant disease (PFI <6 months) following progression on PARPi received subsequent platinum-based chemotherapy; this group had an encouraging ORR of 46.2% and mPFS of 4.7 months. Overall, this body of evidence further draws into question the role of the PFI in determining platinum eligibility, particularly so in the PARPi-resistant setting, and warrants further research.

**Figure 6:**
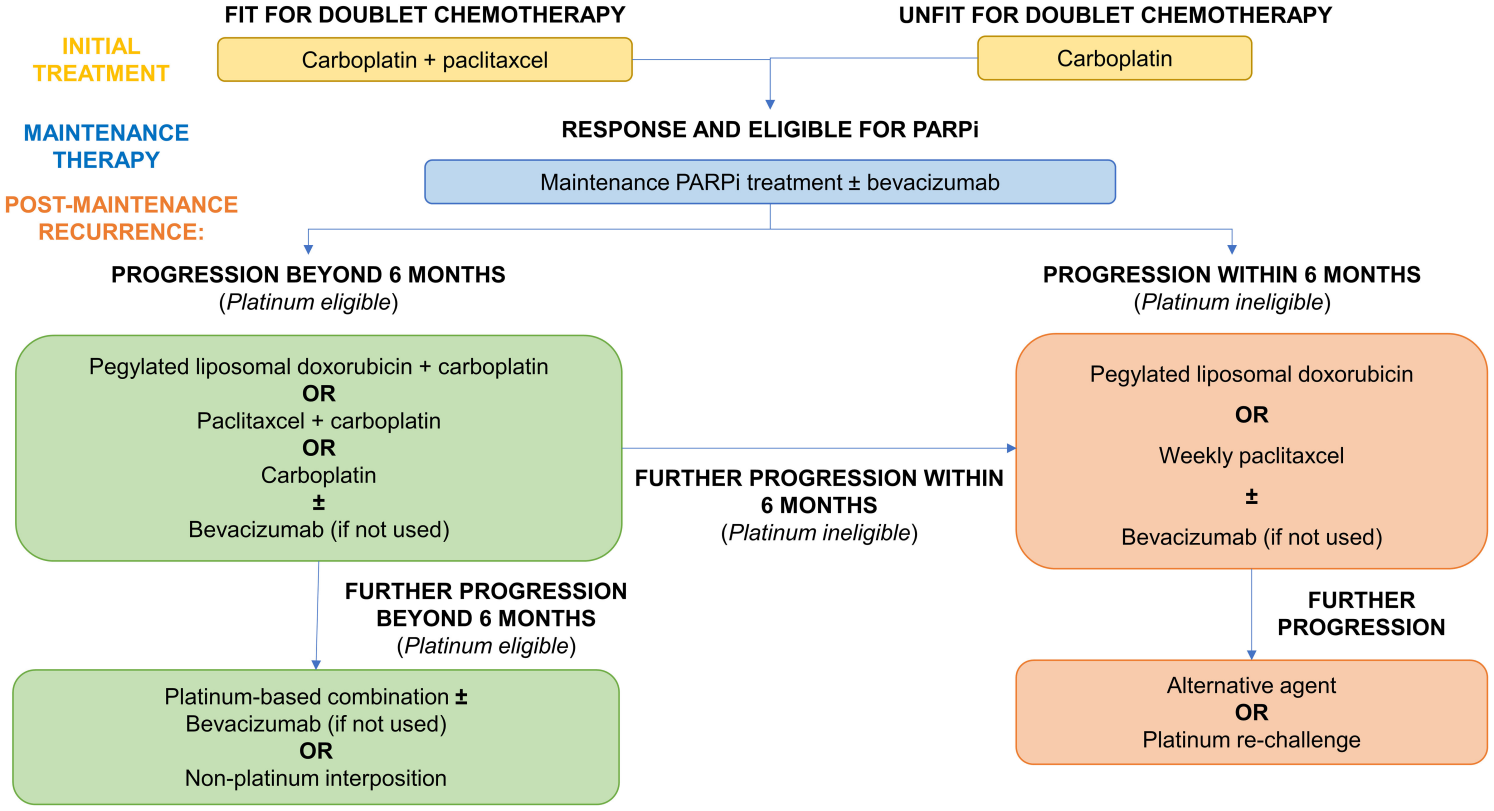
Current treatment algorithm for advanced ovarian cancer patients with progression on PARPi.

The current treatment algorithm for advanced ovarian cancer patients with progression on PARPi is outlined in **Figure 6**, according to platinum-eligibility.

#### 6.1.3 Current management of platinum eligible patients (PFI >6 months)

Beyond rechallenge with platinum-based doublet therapy (165, 175), platinum-eligible patients may also benefit from the addition of anti-angiogenic therapy such as bevacizumab as evidenced across several randomised trials in this setting (176–179). Its benefits in combination with platinum agents may be further realised in PARPi-resistant patients, given its potential to overcome cross-resistance, and warrants further research.

As raised by Caruso et al., further consideration should be given to the potential benefits of non-platinum chemotherapy in those with a PFI of 6-12 months in the post-PARPi setting (165). Despite being labelled as platinum-eligible, interposing non-platinum therapies may overcome cross-resistance but also improve response to later lines of platinum-based therapy. Although this strategy did not improve overall survival in the MITO-8 trial (including patients with a PFI of 6-12 months), it is unclear whether this will hold true following PARPi therapy (180).

#### 6.1.4 Current management of platinum ineligible patients (PFI >6 months)

Typical response rates to non-platinum monotherapy in platinum ineligible patients range from 10-15% (181). In contrast, platinum rechallenge in PARPi-resistant patients with PFI <6 months yielded an ORR of over 40% (174). Therefore, consideration should be given to trialling platinum rechallenge more formally in this specific patient group. Bevacizumab provides additional benefits to progression free survival and is therefore now the standard of care in this group. This was based on the AURELIA trial although bevacizumab appeared to also potentiate chemotherapy toxicity (182).

### 6.2 Predictive biomarkers of PARPi sensitivity and acquired resistance

PARPi resistance can be classified into intrinsic, wherein progression occurs during PARPi maintenance therapy, or acquired in which relapse occurs following completion of PARPi therapy. Clinicopathological factors associated with failure of PARPi therapy include: high pre-treatment serum Ca-125 levels, use of neoadjuvant chemotherapy, non-high grade serous histology and the absence of BRCA mutations (183).

#### 6.2.1 Existing predictors of PARPi sensitivity

As outlined above, the overlapping impacts of platinum agents and PARPi leads to multiple mechanisms of cross-resistance. Consequently, platinum sensitivity may be a proxy marker of PARPi sensitivity. Whilst there is an association regardless of BRCA status, platinum sensitivity holds greater predictive value in non-BRCA mutated tumours as demonstrated across several clinical trials (184, 185). Nonetheless, platinum sensitivity is an unreliable predictor overall; partially restored functionality of the HR pathway may confer PARPi resistance whilst retaining vulnerability to ICLs and other platinum-induced DNA damage (186).

In current clinical practice, PARPi sensitivity is best predicted by identification of pathological BRCA mutations or HRD. Commercially available tests, such as the BRACAnalysis CDx (Myriad Genetics) and FoundationFocus CDxBRCA (Foundation Medicine), utilise next generation sequencing to identify single nucleotide variants or short indels in the BRCA genes. However, larger scale structural variants may also disrupt BRCA1/2 function thereby conferring HRD (187); detection of such changes using whole genome or long read sequencing can improve accuracy in predicting PARPi sensitivity (188).

Pathological detection of HRD can be achieved in three main ways: (1) Next Generation Sequencing of germline mutations in blood lymphocytes, (2) testing for somatic mutations in tumour samples and (3) assessing for genomic instability or mutational scars caused by HRD. The latter results from an over-reliance on more error-prone pathways which manifests as instability signature profiles. These signatures encompass genomic patterns of Loss of Heterozygosity (gLOH), telomeric imbalances, and large-scale transitions which are combined to form a validated HRD score predictive of PARPi sensitivity (189). However, these commercially available tests, such as MyChoice HRD (Myriad Genetics) and FoundationFocus CDxBRCA LOH (Foundation Medicine), cannot identify evidence of restored HR. The inability to detect reversion mutations and epigenetic modifications means these tests are unsuitable for confirming the development of acquired resistance (187). Moreover, whilst PARPi are more effective in BRCA-mutated or HRD tumours, they still demonstrated significant efficacy in HR-proficient tumours across numerous clinical trials (190). There are several potential reasons for this finding. Firstly, current tests may fail to accurately detect the presence of HRD based on the current panel of HR genes tested and mutational scar signatures. Further evaluation of other potential HR-related genes and the role of larger scale structural variants and genomic changes as candidate biomarkers for PARPi sensitivity is necessary. Secondly, PARPi may have wider mechanisms of action beyond BRCA/HRD-related synthetic lethality. Their effects on replication fork stability (67), ribosome biogenesis, transcription of genes (191) and interactions with the immune system (192) warrant further exploration.

#### 6.2.2 Novel predictors of acquired PARPi resistance

Identifying actionable biomarkers of acquired resistance to PARPi is an important next frontier in personalised ovarian cancer therapeutics. Progress has been made in this area however. Firstly, BRCA reversion mutations (as described above) can be detected using next generation sequencing of circulating cell-free DNA (cfDNA) (188). From data in the ARIEL2 trial, pre-treatment reversion mutations in BRCA1/2 (identified using cfDNA) were associated with significantly reduced progression free survival as compared to those without (mPFS 1.8 vs. 9.0 months) (193). This suggests a role for cfDNA in stratifying patients particularly in future clinical trials in the post-PARPi setting. Moreover, the study identified eight patients who acquired BRCA reversion mutations using cfDNA. These patients still derived initial benefit from PARPi therapy although the temporality of reversion mutation acquisition varied. In half of the cases, mutations were detected prior to progression (ranging from 0.7 to 8.3 months prior) whilst in the remainder, mutations were only identified at the time of progression (193). Further data from the EVOLVE trial demonstrated a sensitivity of 74.4% for cfDNA testing as compared to sequencing of tumour tissue. In addition, the fragmentation profile of circulating tumour DNA at baseline was associated with PFS, suggesting a wider prognostic benefit of this tool (194).

As reviewed by Funingana et al. (188), advancements in cfDNA testing such as targeted sequence approaches, capture hybridisation-based methods and shallow whole genome sequencing have markedly improved its sensitivity. Interestingly, the latter has been used to sequence cfDNA from dried blood spots thus demonstrating potential to be developed into finger prick tests for longitudinal monitoring of patients (195, 196). However, questions remain over the availability of cfDNA within ascitic and pleural samples as well as the sensitivity for reversion mutations (188). The findings from ongoing observational studies and incorporation in the next generation of clinical trials (188) are essential for validation and will help to define the clinical utility of cfDNA testing for reversions.

Furthermore, the clinical utility of testing for the various mechanisms of acquired PARPi resistance remains unclear. For instance, evaluation of SLFN11 expression by immunohistochemistry is now clinically feasible and may be used to identify acquired resistance (140). Similarly, novel functional assays of HRD may also offer a means of better predicting PARPi sensitivity as reviewed in detail by Arcieri et al. (197) Immunofluorescence testing to measure RAD51 foci formation has been validated as a PARPi biomarker in pre-clinical studies (122, 198), although the predictive value of assessing epigenetic modifications remains unclear (199). Finally, a novel approach of using PET imaging to quantify regional expression of PARP1 has shown to potential to act as a non-invasive biomarker of PARPi resistance (200).

The extent to which these assays can guide future treatment decisions relies heavily on gathering further evidence from post-PARPi trials. Moreover, it should be acknowledged that intra-tumoral heterogeneity, subclonality and the multifactorial nature of resistance mechanisms to PARPi will hinder efforts to identify novel biomarkers and potential targets.

## 7 Strategies to bypass PARPi resistance

Following progression on or after PARPi therapy, employing alternative therapeutic strategies to bypass resistance mechanisms must be explored, either as monotherapy or in combination with PARPi. These include immunotherapeutics, antibody drug conjugates (ADCs) and modulation of glucocorticoid and receptor tyrosine kinase signalling.

### 7.1 Immunotherapy

Ovarian cancer can be considered an immunogenic pathology; antitumour immune responses have been detected as well as a specific immunoreactive molecular subtype associated with longer overall survival (201). In particular, the presence of CD8+ and CD20+ tumour infiltrating lymphocytes (TILs) have been shown to confer a better prognosis in ovarian cancer (202). Nevertheless, ovarian cancers demonstrate an immunosuppressive tumour microenvironment as a result of regulatory T (Treg) cells, tumour associated macrophages, myeloid-derived suppressor cells, cancer-associated fibroblasts and adipocytes. The formation of immunosuppressive networks results in suppression of CD8+ TILs by Treg cells and increased expression of inhibitory receptors such as programmed cell death protein 1 (PD-1) and cytotoxic T-lymphocyte-associated protein 4 (CTLA-4) (203, 204). This poses a significant challenge to the efficacy of immunotherapeutic strategies.

#### 7.1.1 Immune checkpoint inhibitors

Monoclonal antibodies which block immune checkpoints expressed on T cells, such as PD-1 and CTLA-4, or tumour cells, such as programmed death ligand 1 (PD-L1), have demonstrated effectiveness across a wide range of solid tumours (204). However, their effectiveness in ovarian cancer has been limited for the reasons outlined above. The Javelin Ovarian 100 (205) and 200 (206) trials examined the effectiveness of avelumab (PD-L1 antibody) in combination with chemotherapy in the first-line and recurrent setting. Neither study demonstrated significant improvements in PFS. Likewise, negligible benefits in PFS have been observed with atezolizumab (PD-L1 antibody). In a phase III, placebo-controlled randomised trial (IMagyn050), immune checkpoint inhibition improved mPFS by 1 month over placebo in the overall population (19.5 vs. 18.4 months) and 2 months in the PD-L1-positive subgroup (20.8 vs. 18.5 months) (207). Similar results were observed in the platinum-sensitive recurrent setting in the ATLANTE trial comparing atezolizumab to placebo with chemotherapy and bevacizumab (overall population mPFS: 13.5 vs 11.2 months; PD-L1-positive group: 15.2 vs. 13.1 months) (208).

On the other hand, combinations of immune checkpoint inhibitors with PARPi have demonstrated synergism and clear clinical benefit. PARP inhibition may carry wide ranging immunostimulatory effects. The propagation of DNA damage causes the release of cytosolic DNA which in turn activates the cyclic GMP-AMP synthase (cGAS)-stimulator of interferon genes (STING) pathway. This not only occurs within the tumour cells but, following exocystosis of cytosolic DNA, also results in activation of the pathway in neighbouring dendritic cells in a paracrine fashion. Consequently, STING pathway activation culminates in a type 1 interferon response and enhanced antigen presenting ability. Moreover, PARPi increases the susceptibility of tumour cells to natural killer (NK) cell mediated apoptosis, encourages pro-inflammatory differentiation of T cells and down-regulation of immune checkpoint receptors such as PD-1. However, PARP inhibition also results in up-regulation of PD-L1 expression and therefore immunosuppression. These interactions between PARP inhibition and the immune system are reviewed in detail in (192). This pre-clinical basis has rationalised several phase I and II trials of immune checkpoint and PARP inhibitors which have demonstrated encouraging success (209–211).

Concerns have been raised however, regarding whether the immunogenic effects of PARP inhibition, such as STING activation, are reliant on the presence of HRD (152). If this is the case, then such combinations are unlikely to be successful in the context of restored HR proficiency and PARPi resistance. Encouragingly however, evidence from early phase trials suggest patients may still derive similar benefits regardless of HRD status (210, 212). Most recently, data from the phase III, randomised, placebo-controlled DUO-O trial demonstrated the significant benefits of durvalumab in combination with chemotherapy and bevacizumab and followed by maintenance durvalumab, bevacizumab and olaparib (213). This treatment approach significantly improved mPFS as compared to placebo (37.3 vs. 23.0 months), notably across both the HRD and HR proficient subgroups. Trials of immunotherapy approaches in the PARPi-resistant setting, as both monotherapy and in combinations, are essential moving forward.

#### 7.1.2 Novel immunotherapeutic agents

Other novel immunotherapy strategies in ovarian cancer include the use of engineered cytokines, such as nemvaleukin alfa which binds to the intermediate affinity interleukin-2 (IL-2) receptor. This preferentially activates CD8+ T and NK cells over Treg cells and minimises adverse effects by not binding to the high affinity receptors (203). The ARTISTRY-1 trial, which tested the drug in combination with pembrolizumab, demonstrated durable antitumour activity across solid tumours, including platinum-resistant ovarian cancer (214). Other novel therapies in the early stages of development include bispecific antibodies, such as ubamatamab which targets MUC16/CD3 to promote T cell cytotoxicity (215), and T cell activating vaccines such as maveropepimut-S (216).

#### 7.1.3 Chimeric antigen receptor T cell therapy

Allogeneic CAR-T cells are produced by genetic modification of autologous T cells ensuring the expression of a tumour antigen-specific CAR. The cell population is expanded *ex vivo* prior to reinfusion into the patient (217). The CAR-T cell itself is produced through combining the single-chain variable region (scFv) of the monoclonal antibody and the T-cell coreceptors signalling region. The scFv within the CAR directly activates the T cell after binding to its complementary tumour-specific antigen (TSA) leading to cell death, independent of MHC expression (218). Unfortunately, implementing CAR-T cell therapy in ovarian cancer is fraught with difficulties. Firstly, ovarian cancer often lacks TSAs and therefore the treatment must target a broad range of antigens. Moreover, CAR-T cell therapy must overcome issues such as off-target effects (leading to potentially serious adverse events), tumour antigen escape (such as loss or downregulation of TSAs) and heterogeneity, as well as the immunosuppressive tumour microenvironment. The challenges and potential strategies to overcome these are reviewed in detail in (218). Encouraging evidence has been observed in a phase I trial for recurrent ovarian cancer which certainly warrants further investigation (219).

#### 7.1.4 Oncolytic virus therapy

Oncolytic viruses are a ‘living’ therapy which offer a novel approach to treating ovarian cancer. These viruses specifically infect and kill tumour cells during their replication process, in turn releasing large numbers of progeny virions which can attack further tumour cells. Their specificity for neoplastic host cells relies on either: (1) selective uptake due to changes in the viral envelope, (2) absence or loss of function of a gene which is necessary for replication in normal cells but not in tumour cells or (3) use of tumour-specific promoters to regulate viral gene expression (220). Both vaccine and tumour-selective genetically engineered viruses have demonstrated promising efficacy in early phase trials as reviewed in detail in (221). Whilst such therapies can be delivered locally into the peritoneal cavity, it faces similar issues to other immunotherapeutics, namely tumour heterogeneity and the immunosuppressive microenvironment. Trialling combination therapy with immune checkpoint inhibitors or CAR-T cells may overcome these issues in the future (222).

### 7.2 Antibody drug conjugates

ADCs offer a novel targeted approach in the treatment of ovarian cancer by conjugating cytotoxic agents to monoclonal antibodies specific to cancer cells. Binding of the antibody results in internalisation of the cytotoxic agent to tumour cells alone thereby minimising systemic toxicity. One example is mirvetuximab soravtansine which targets folate receptor-α (FR-α); the soravtansine component is a microtubule inhibitor. This ADC has been investigated in the FORWARD-1 trial which included patients with platinum-resistant disease and positive FR-α expression. However, there was no significant improvement in mPFS as compared to chemotherapy alone (4.1 vs. 4.4 months) (223). In platinum-sensitive patients on the other hand, the ADC combination with bevacizumab and carboplatin demonstrated high activity in FR-α positive patients (224).

Interestingly, the single-arm phase II SORAYA trial of the same ADC specifically explored the efficacy in platinum-resistant patients with prior PARPi exposure. The ORR was 38.0% (95% CI: 24.7-52.8) in those with prior PARPi compared to 27.5% (95% CI: 15.9-41.7) in those without (225). The difference in findings may relate to differing estimation criteria for FR-α expression (203). Overall, this suggests a potential role in this population dependent on careful stratification by FR-α expression.

Anti-NaPi2b ADCs such as lifastuzumab vedotin (226) and upifitamab rilsodotin (227) target the sodium-dependent phosphate transport protein which is highly specific for ovarian tumour cells over normal tissue (203). Other targeted proteins include tissue factor (by tisotumab vedotin) (228) and mesothelin (by anetumab ravtansine) (229). These agents have shown encouraging anti-tumour activity in early phase trials, albeit without evidence of improvements in PFS. It is likely that delivery of ADCs is hampered by the same issues as immunotherapeutic agents as described above.

Use of ADCs may overcome the toxicity issues which plague combinations of PARPi and chemotherapy. For instance, sacituzumab govitecan targets TROP2 (commonly overexpressed in ovarian cancer) and works synergistically with PARPi as well as overcoming resistance in PARPi-resistant cell lines (230). A phase I trial of the ADC with rucaparib demonstrated encouraging anti-tumour activity in patients with prior PARPi exposure although dose-limiting toxicity remained an issue (231). Nevertheless, this marks an important development in such combination therapies and trials of pulsed-dosing regimens may yield better toxicity profiles.

### 7.3 Selective glucocorticoid receptor modulators

Cortisol acts to suppress apoptotic pathways activated by chemotherapy agents and thus contributes to treatment resistance. Relacorilant modulates glucocorticoid receptors, which are widely expressed on ovarian cancer cells, to reverse this anti-apoptotic effect and re-sensitise tumour cells to chemotherapy (232). A randomised, open-label phase II trial investigated combination therapy (with intermittent relacorilant) compared to chemotherapy alone in platinum-resistant disease. Whilst the study demonstrated significant improvements in mPFS (5.55 vs. 3.76 months) (233), there was no significant improvement in overall survival (234). The association between glucocorticoid receptor expression and PARPi resistance may warrant further investigation as it may rationalise treatment with relacorilant in the resistant setting.

### 7.4 Gas6/Axl signalling

Growth arrest specific 6 (Gas6) binds to Axl, a receptor tyrosine kinase which is specifically expressed on ovarian cancer cells over normal cells (235). Binding of Gas6 results in signalling pathways promoting cellular proliferation and survival, resulting in an association with chemoresistance and inferior patient outcomes (203). Batiraxcept is a novel therapeutic which acts as an Axl decoy receptor with far greater affinity for Gas6; it demonstrated encouraging activity when administered in combination with paclitaxcel during a phase Ib study (236). However, a phase III, placebo-controlled trial of this combination found a lower progression free survival as compared to paclitaxcel plus placebo (5.1 vs. 5.5 months) and was therefore terminated (NCT04729608) (237).

## 8 Targeting the DDR to overcome PARPi resistance

Targeting of the DNA damage response (DDR), either towards alternative pathways, drivers of cell cycle progression or novel synthetically lethal pairings, can offer another potential means of overcoming PARPi resistance.

### 8.1 PARPi rechallenge

#### 8.1.1 PARPi rechallenge as monotherapy

At present, the evidence base for PARPi rechallenge is largely limited to the phase III OReO trial in platinum-sensitive recurrent ovarian cancer patients who had previously received at least 6 months of maintenance PARPi (81). Patients were not tested for reversion mutations or functional assays of HRD, rather stratification was based on previously documented BRCA status and HRD testing of archival tissue. Compared to placebo across 220 patients, olaparib rechallenge significantly improved mPFS in both the BRCA-mutated (4.3 vs. 2.8 months) and non-BRCA-mutated cohorts (5.3 vs. 2.8 months). PARPi rechallenge also appeared to demonstrate some benefit even in HRD-proficient patients in this setting although statistical significance was not reached (likely due to the small sample size). Overall, the trial demonstrated the significant benefits of PARPi rechallenge in platinum-sensitive patients; mature OS data is necessary to determine the longevity of these responses. Smaller scale real-world data from retrospective studies re-treated with further PARPi supports these findings, particularly where patients meet the OReO inclusion criteria (238, 239).

Across these studies, a small proportion of patients received a different PARPi to their prior therapy. Within the OReO study, this was most commonly a move to olaparib from either niraparib or rucaparib in non-BRCAm patients (given their FDA licensed indications in **Table 1**) (81). The impact of rechallenging with a different PARPi agent may warrant further exploration given the benefits observed in this subgroup and the variability of trapping potency amongst PARPi’s. For instance, use of newer PARPi’s such as pamiparib may result in better outcomes following rechallenge due to their superior potency over olaparib (240). Comparative trials of different PARPi agents in the rechallenge setting may be an important next step.

#### 8.1.2 PARPi rechallenge in combination therapies

Consideration has been given to the role of PARPi rechallenge in combination with locoregional therapies including surgery or radiotherapy. In theory, such treatments can remove the treatment-resistant clones leaving sensitive tumour cells amenable to treatment. The benefits of this approach have been demonstrated in two retrospective studies of women with BRCA-mutated platinum sensitive recurrent disease; secondary cytoreductive surgery prior to platinum re-treatment and olaparib maintenance significantly improved patient outcomes (241, 242). However, PARPi was only commenced in the second line setting in both studies. Secondary cytoreductive surgery may carry greater effectiveness in the primary post-PARPi setting through removal of PARPi-resistant clones. This rationalises the ongoing phase III MITO 35b trial investigating the effectiveness of using olaparib beyond progression following secondary cytoreductive surgery in patients who have received previous PARPi (243).

As outlined above, anti-angiogenic agents down-regulate HR genes such as BRCA1/2 and RAD51 through inducing hypoxia in the tumour microenvironment and interactions with other transcriptional repressors (77). It therefore follows that anti-angiogenic therapy with agents such as cediranib can sensitise tumours to PARPi (244) and even potentially overcome acquired resistance. The phase II EVOLVE study evaluated this combination in a cohort of 34 patients who had progressed on PARPi. Overall, no objective responses were seen in platinum sensitive patients and just 2 of 10 platinum resistant patients had an objective response. More importantly, although limited by a small sample size, the study demonstrated significantly inferior responses for patients with confirmed HR reversion and up-regulation of the ABCB1 transporter (114). This highlights the potential benefits of identifying acquired resistance mechanisms to guide treatment decisions.

Combinations of PARPi and chemotherapy are often hindered by overlapping toxicity profiles, in particular with regards to myelosuppression (1). Currently licensed PARPi inhibit both PARP1 and PARP2; inhibition of the former is thought to drive synthetic lethality whilst the latter may be more associated with haematological toxicity (245). The newly developed selective PARP1 inhibitor, AZD5305, has shown efficacious responses in pre-clinical settings and in the phase I PETRA trial (NCT04644068) (245–247). If the reduced risk of myelosuppression translates in the clinical setting, selective PARP1 inhibition may offer a means of optimising combination therapies in both the primary and resistant settings.

### 8.2 ATR/CHK1 inhibition

**Figure 7:**
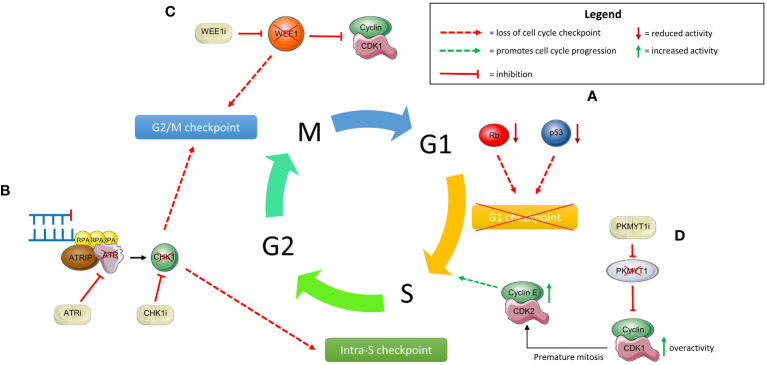
Targeting cell cycle checkpoints and exploiting synthetic lethality through the DNA damage response (DDR). **(A)** Loss of Rb and p53 result in loss of a functional G1 checkpoint thus rendering cells susceptible to synthetic lethality strategies which cause loss of other checkpoints. **(B)** One example of this is ATR inhibition (ATRi) or CHK1 inhibition (CHK1i); this results in loss of both the intra-S and G2/M checkpoints. In the context of loss of the G1 checkpoint as in **(A)**, this results in synthetic lethality. **(C)** Similarly, inhibition of WEE1 with small molecule inhibitors (WEE1i) prevents inhibition of CDK1 and hence loss of the G2/M checkpoint. This results in synthetic lethality through the same mechanism. **(D)** Cyclin E overexpression can be targeted through inhibition of PKMYT1. Inhibition of PKMYT1 (PKMYTi) results in loss of inhibition of CDK1 and hence its overactivity. Coupled with overexpressed cyclin E results in premature mitotic entry, mitotic catastrophe and hence synthetic lethality.

As shown in **Figure 7**, components of the DDR which govern cell cycle checkpoints may offer alternative synthetic lethality targets.

The ataxia telangiectasia and Rad3-related (ATR)-checkpoint kinase 1 (CHK1) pathway is integral within the DDR through activation of the intra-S and G2/M cell cycle checkpoints as discussed in section 5. Therefore, cells lacking a functional G1 checkpoint, such as those with p53 mutations, may be liable to synthetic lethality through ATR/CHK1 inhibition. Notably, driver mutations in p53 are ubiquitous in HGSOC suggesting they may be particularly sensitive to ATR inhibition (248). In these cells, ATR or CHK1 inhibition results in loss of the G1, intra-S and G2/M checkpoints with premature progression to mitosis leading to a ‘mitotic catastrophe’ (2). Moreover, loss of 53BP1, which is thought to contribute to PARPi resistance, was shown to exhibit strong synthetic lethality with an ATR inhibitor and cisplatin combination therapy (249). In the pre-clinical setting, use of ATR/CHK1 inhibitors in combination with olaparib overcame olaparib-resistant in BRCA2-mutated ovarian cancer cell lines (250).

Several small-molecule ATR inhibitors have been evaluated in clinical trial thus far with the most developed being M6620 (berzosertib, IV) and AZD6738 (ceralasertib, PO) (2). As reviewed in more detail in (251), ATR inhibitors have shown significant improvements in mPFS over chemotherapy alone in early phase trials (252, 253). Whilst these data suggest a potential role for ATR inhibitors in treating PARPi-resistant patients, further development of predictive biomarkers of sensitivity to the therapy is key. Potential markers include proteins involved in DNA synthesis, such as DNA polymerases, functional assays of HRD, replication stress markers and proteins involved at other cell cycle checkpoints (251). Further elucidating potential mechanisms of resistance to ATR inhibition, such as loss of cyclin C or CDK8, is also an important next step in their development as a targeted therapy – particularly so in the context of patients already resistant to PARPi (254).

Selective CHK1 inhibitors have also been recently developed, such as MK-8776, with encouraging evidence as monotherapy and in combination during pre-clinical studies (255, 256). Prexasertib is a second-generation CHK1 inhibitor which also possesses anti-CHK2 activity. Two phase II trials of prexasertib monotherapy demonstrated its clinical activity although neutropenia was common in both (257, 258). In one trial, 41 patients were recruited with BRCA-mutated platinum-resistant disease who had progressed on PARPi therapy; this group had an ORR of 12.2% with prexasertib therapy (258). Furthermore, in p53-deficient tumours such as HGSOC, pre-clinical evidence suggests CHK1 inhibition may induce an HRD phenotype and thus sensitise cells to PARPi (259). Evidently, the extent to which this applies in the PARPi-resistant setting is unclear but it may rationalise further trials of PARPi/CHK1 inhibitor combinations.

### 8.3 WEE1 inhibition

In a similar manner to ATR, WEE1 kinase plays a critical role in controlling cell cycle progression at the G2/M checkpoint as described above. Therefore, loss of a functional G1/S checkpoint also renders cells sensitive to WEE1 kinase inhibition. Consequently, several WEE1 inhibitors have been utilised in trials, the most developed of which is adavosertib (260). This drug has been tested in numerous phase II trials for advanced ovarian cancer yielding encouraging results (261–264). Interestingly in one study, CCNE1 amplification was associated with greater benefit from WEE1 inhibition although SLFN11 levels were not predictive of response (264). This was further highlighted in the CCNE1-stratified IGNITE trial of recurrent, platinum-resistant ovarian cancer; the ORR was 38% in over-expressed and amplified tumours and 45% in over-expressed only tumours (265). This suggests a potential role for WEE1 inhibition in PARPi-resistant patients secondary to CCNE1 overexpression. Finally, the EFFORT trial compared the efficacy of adavosertib monotherapy to its combination with olaparib in 80 ovarian cancer patients who had progressed on PARPi. Both approaches demonstrated efficacy (ORR 23% with adavosertib alone and 29% in combination) with no significant differences in mPFS (5.5 and 6.8 months respectively) (266). Such a combination takes advantage of both drugs’ effects on the DNA damage response and highlights the potential benefits of this approach. Taken together, the evidence supports the need for larger scale trials evaluating WEE1 inhibitors in the PARPi-resistant setting, although its use may be limited by its toxicity profile (267).

### 8.4 PKMYT1 inhibition

Whilst WEE1 inhibition may be a useful means of treating tumours with CCNE1 overexpression, identifying novel synthetic lethality targets through genome wide screens may highlight alternative strategies. The discovery of a synthetic lethal interaction between PKMYT1 and CCNE1 amplification is a pertinent example of this (268). As described above, CCNE1 amplification is associated with PARPi resistance and is mutually exclusive with HRD (114, 135). CCNE1 plays vital roles in cell cycle progression, predominantly through activation of cyclin dependent kinase 2 (CDK2) resulting in downstream phosphorylation of RB1 and transition from G1 to S phase (269). PKMYT1 encodes a protein kinase, closely related to WEE1 kinase, which inhibits activity of CDK1 through phosphorylation and sequestration in the cytoplasm. CCNE1 amplification drives DNA replication stress and transcription of the MMB-FOXM1 complex. In turn, this complex increases levels of cyclin B and CDK1 during S phase. Inhibition of PKMYT1 results in overactive CDK1 and therefore premature progression to mitosis, eventually leading to mitotic catastrophe (268). A novel PKMYT1 inhibitor RP-6306 (lunresertib) has been developed and is currently being evaluated in early phase clinical trials (270). Given the exclusive association between CCNE1 overexpression and HR proficiency, this drug may have particular efficacy in the PARPi-resistant population.

### 8.5 Polθ inhibition

As outlined above, up-regulation of POLQ may be responsible for driving PARPi resistance although the precise mechanism remains to be elucidated. Nevertheless, polθ inhibitors, such as novobiocin, RP-6685 and ART558, have been identified or developed (271). The latter has been shown to reverse PARPi resistance secondary to alterations in 53BP1 or the Shieldin complex (147). This finding has been further supported across many *in vitro* studies; however, ART558 has been shown to have a low *in vivo* metabolic stability in rat microsomes which may hinder its translation to clinical trials (271).

### 8.6 Targeting RAS/MAPK/MEK

Pre-clinical evidence suggests that RAS mutations may confer resistance to PARPi. Moreover, *in vivo* evidence suggests synergism between inhibition of MEK, downstream of RAS, and PARPi (272, 273). The benefits of MEK inhibition may lie in remodelling of the immune response and microenvironment; inhibition of the RAS pathway with such drugs resulted in STING pathway activation, CD8+ T-cell recruitment and reduced myeloid-derived suppressor cell infiltration (274). In addition, MEK inhibition may reduce capacity for HR repair (1). Therefore, this approach may overcome resistance to both PARPi and immune checkpoint inhibitor resistance and warrants further exploration, possibly through combination treatments (272).

### 8.7 G-Quadruplex stabilisers

Whilst DNA normally adopts a canonical right-handed helix (B-DNA), it can fold into alternative structures including G-quadruplexes (G4) (275). These four-stranded helical structures impede DNA polymerases and repair processes thereby contributing to genomic instability (276). Repair of G4-induced DNA damage is predominantly via HR repair (277). Consequently, use of G4-stabilising drugs may result in synthetic lethality in HRD tumours due to accumulation of unrepaired damage and replication fork arrest (203). A phase I trial of pidnarulex (CX-5461), a G4-stabiliser, demonstrated promising anti-tumour efficacy in patients with HRD solid tumours (ORR 14%). However, reversion mutations in PALB2 and BRCA2 resulted in resistance to G4-stabilising therapy which may suggest limited utility in the PARPi-resistant population where this is the underlying mechanism of resistance (278).

### 8.8 Epigenetic resensitisation

As discussed above, epigenetic changes within key DDR genes may be responsible for the development of acquired PARPi resistance in some patients. Therefore, epigenetic modulators such as DNA methyltransferase (DNMT) and histone deacetylase (HDAC) inhibitors may help in preventing the development of treatment resistance (169). Moreover, DNMT inhibitors may induce HRD by altering expression of DSB repair genes which further rationalises future trials of DNMT and PARP inhibitor combinations (279). Early phase trials support the use of DNMT and HDAC inhibitors in combination with chemotherapy for ovarian cancer but their benefit in acquired PARPi-resistance is unclear (280, 281).

A novel epigenetic approach is through the inhibition of BET proteins. BET proteins play critical roles in initiating and continuing transcription as well as cell cycle regulation (282). By binding to the bromodomains of BET proteins, inhibitors can prevent interaction with acetylated histones and transcription factors thus reducing expression of repair proteins such as BRCA1 and RAD51 (283). BET inhibitors may therefore induce an HRD phenotype hence rationalising combination treatment with PARPi and offering a novel means of overcoming resistance (283, 284).

## 9 Conclusion

The DNA damage signaling response and repair (DDR) is a critical defense mechanism against genomic instability. Discovery of anti-cancer drug targets within DDR has rapidly advanced to clinically viable drugs for ovarian cancer patients. Such a precision oncology strategy is best exemplified by the current clinical use of PARP inhibitors in BRCA germ-line deficient and platinum sensitive sporadic epithelial ovarian cancers. Whilst tremendous advances in precision oncology strategies have improved patient outcomes, the development of intrinsic or acquired resistance to such therapies remains a formidable clinical challenge and limits survival. The development of predictive biomarkers including the evaluation of cfDNA as a tool to detect emergence of early resistance will likely provide further insights soon. Surgical salvage and the development of other targeted therapies (such as those targeting ATM, ATR, WEE1 and others) may address this unmet clinical need. Finally, discovery of additional synthetic lethality interaction partners focused on DDR remains an area of intense investigation and will help advance in precision medicine in ovarian cancer. Next generation of DNA repair inhibitors either as monotherapy or in combination with PARP inhibitors could potentially improve outcomes but will need to be tested in phase III randomized trials in ovarian cancer.

## Author contributions

SK: Conceptualization, Data curation, Formal analysis, Methodology, Writing – original draft, Writing – review & editing, Visualization. KG: Conceptualization, Data curation, Formal analysis, Writing – original draft, Writing – review & editing. SM: Conceptualization, Data curation, Formal analysis, Writing – original draft, Writing – review & editing, Funding acquisition, Investigation, Methodology, Project administration, Resources, Software, Supervision, Validation, Visualization.

## References

[1] Bhamidipati D Haro-Silerio JI Yap TA Ngoi N. PARP inhibitors: enhancing efficacy through rational combinations. Br J Cancer. (2023) 129:904–16. doi: 10.1038/s41416-023-02326-7 PMC10491787 37430137

[2] Kulkarni S Brownlie J Jeyapalan JN Mongan NP Rakha EA MadhuSudan S. Evolving DNA repair synthetic lethality targets in cancer. Biosci Rep. (2022) 42. doi: 10.1042/BSR20221713 PMC9760629 36420962

[3] Brownlie J Kulkarni S Algethami M Jeyapalan JN Mongan NP Rakha EA. Targeting DNA damage repair precision medicine strategies in cancer. Curr Opin Pharmacol. (2023) 70:102381. doi: 10.1016/j.coph.2023.102381 37148685

[4] Huang J Chan WC Ngai CH Lok V Zhang L Lucero-Prisno DE 3rd. Worldwide burden, risk factors, and temporal trends of ovarian cancer: A global study. Cancers (Basel). (2022) 14:2230. doi: 10.3390/cancers14092230 35565359 PMC9102475

[5] Dilley J Burnell M Gentry-Maharaj A Ryan A Neophytou C Apostolidou S. Ovarian cancer symptoms, routes to diagnosis and survival - Population cohort study in the 'no screen' arm of the UK Collaborative Trial of Ovarian Cancer Screening (UKCTOCS). Gynecol Oncol. (2020) 158:316–22. doi: 10.1016/j.ygyno.2020.05.002 PMC7453382 32561125

[6] Baldwin LA Huang B Miller RW Tucker T Goodrich ST Podzielinski I. Ten-year relative survival for epithelial ovarian cancer. Obstet Gynecol. (2012) 120:612–8. doi: 10.1097/AOG.0b013e318264f794 22914471

[7] Morgan RD Clamp AR Barnes BM Timms K Schlecht H Yarram-Smith L. Homologous recombination deficiency in newly diagnosed FIGO stage III/IV high-grade epithelial ovarian cancer: a multi-national observational study. Int J Gynecol Cancer. (2023) 33:1253–9. doi: 10.1136/ijgc-2022-004211 37072323

[8] Tang H Kulkarni S Peters C Eddison J Al-Ani M MadhuSudan S. The current status of DNA-repair-directed precision oncology strategies in epithelial ovarian cancers. Int J Mol Sci. (2023) 24:7293. doi: 10.3390/ijms24087293 37108451 PMC10138422

[9] Li H Liu ZY Wu N Chen YC Cheng Q Wang J. PARP inhibitor resistance: the underlying mechanisms and clinical implications. Mol Cancer. (2020) 19:107. doi: 10.1186/s12943-020-01227-0 32563252 PMC7305609

[10] Dilmac S Ozpolat B. Mechanisms of PARP-inhibitor-resistance in BRCA-mutated breast cancer and new therapeutic approaches. Cancers (Basel). (2023) 15:3642. doi: 10.3390/cancers15143642 37509303 PMC10378018

[11] Groelly FJ Fawkes M Dagg RA Blackford AN Tarsounas M. Targeting DNA damage response pathways in cancer. Nat Rev Cancer. (2023) 23:78–94. doi: 10.1038/s41568-022-00535-5 36471053

[12] Abbotts R Thompson N MadhuSudan S. DNA repair in cancer: emerging targets for personalized therapy. Cancer Manag Res. (2014) 6:77–92. doi: 10.2147/CMAR.S50497 24600246 PMC3933425

[13] Caldecott KW. DNA single-strand break repair. Exp Cell Res. (2014) 329:2–8. doi: 10.1016/j.yexcr.2014.08.027 25176342

[14] Robertson AB Klungland A Rognes T Leiros I. DNA repair in mammalian cells: Base excision repair: the long and short of it. Cell Mol Life Sci. (2009) 66:981–93. doi: 10.1007/s00018-009-8736-z PMC11131461 19153658

[15] Fortini P Pascucci B Parlanti E D'Errico M Simonelli V Dogliotti E. The base excision repair: mechanisms and its relevance for cancer susceptibility. Biochimie. (2003) 85:1053–71. doi: 10.1016/j.biochi.2003.11.003 14726013

[16] Strom CE Johansson F Uhlen M Szigyarto CA Erixon K Helleday T. Poly (ADP-ribose) polymerase (PARP) is not involved in base excision repair but PARP inhibition traps a single-strand intermediate. Nucleic Acids Res. (2011) 39:3166–75. doi: 10.1093/nar/gkq1241 PMC3082910 21183466

[17] Fisher AE Hochegger H Takeda S Caldecott KW. Poly(ADP-ribose) polymerase 1 accelerates single-strand break repair in concert with poly(ADP-ribose) glycohydrolase. Mol Cell Biol. (2007) 27:5597–605. doi: 10.1128/MCB.02248-06 PMC1952076 17548475

[18] de Laat WL Jaspers NG Hoeijmakers JH. Molecular mechanism of nucleotide excision repair. Genes Dev. (1999) 13:768–85. doi: 10.1101/gad.13.7.768 10197977

[19] Shuck SC Short EA Turchi JJ. Eukaryotic nucleotide excision repair: from understanding mechanisms to influencing biology. Cell Res. (2008) 18:64–72. doi: 10.1038/cr.2008.2 18166981 PMC2432112

[20] Spivak G. Nucleotide excision repair in humans. DNA Repair (Amst). (2015) 36:13–8. doi: 10.1016/j.dnarep.2015.09.003 PMC4688078 26388429

[21] Reardon JT Sancar A. Nucleotide excision repair. In: Progress in Nucleic Acid Research and Molecular Biology, vol. 79. Cambridge, Massachusetts: Academic Press (2005). p. 183–235. 16096029 10.1016/S0079-6603(04)79004-2

[22] Kolodner RD Marsischky GT. Eukaryotic DNA mismatch repair. Curr Opin Genet Dev. (1999) 9:89–96. doi: 10.1016/S0959-437X(99)80013-6 10072354

[23] Papadopoulos N Lindblom A. Molecular basis of HNPCC: mutations of MMR genes. Hum Mutat. (1997) 10:89–99. doi: 10.1002/(ISSN)1098-1004 9259192

[24] Li G-M. Mechanisms and functions of DNA mismatch repair. Cell Res. (2008) 18:85–98. doi: 10.1038/cr.2007.115 18157157

[25] Bebenek A Ziuzia-Graczyk I. Fidelity of DNA replication-a matter of proofreading. Curr Genet. (2018) 64:985–96. doi: 10.1007/s00294-018-0820-1 PMC6153641 29500597

[26] Nakamura K Banno K Yanokura M Iida M Adachi M Masuda K. Features of ovarian cancer in Lynch syndrome (Review). Mol Clin Oncol. (2014) 2:909–16. doi: 10.3892/mco.2014.397 PMC4179837 25279173

[27] Brandsma I Gent DC. Pathway choice in DNA double strand break repair: observations of a balancing act. Genome Integr. (2012) 3:9. doi: 10.1186/2041-9414-3-9 23181949 PMC3557175

[28] Blackford AN Jackson SP. ATM, ATR, and DNA-PK: the trinity at the heart of the DNA damage response. Mol Cell. (2017) 66:801–17. doi: 10.1016/j.molcel.2017.05.015 28622525

[29] Karanam K Kafri R Loewer A Lahav G. Quantitative live cell imaging reveals a gradual shift between DNA repair mechanisms and a maximal use of HR in mid S phase. Mol Cell. (2012) 47:320–9. doi: 10.1016/j.molcel.2012.05.052 PMC3494418 22841003

[30] Stinson BM Loparo JJ. Repair of DNA double-strand breaks by the nonhomologous end joining pathway. Annu Rev Biochem. (2021) 90:137–64. doi: 10.1146/annurev-biochem-080320-110356 PMC8899865 33556282

[31] Koch CA Agyei R Galicia S Metalnikov P O'Donnell P Starostine A. Xrcc4 physically links DNA end processing by polynucleotide kinase to DNA ligation by DNA ligase IV. EMBO J. (2004) 23:3874–85. doi: 10.1038/sj.emboj.7600375 PMC522785 15385968

[32] Riballo E Kuhne M Rief N Doherty A Smith GC Recio MJ. A pathway of double-strand break rejoining dependent upon ATM, Artemis, and proteins locating to gamma-H2AX foci. Mol Cell. (2004) 16:715–24. doi: 10.1016/j.molcel.2004.10.029 15574327

[33] Davis AJ Chen DJ. DNA double strand break repair via non-homologous end-joining. Transl Cancer Res. (2013) 2:130–43. doi: 10.3978/j.issn.2218-676X.2013.04.02 PMC3758668 24000320

[34] Wang C Lees-Miller SP. Detection and repair of ionizing radiation-induced DNA double strand breaks: new developments in nonhomologous end joining. Int J Radiat Oncol Biol Phys. (2013) 86:440–9. doi: 10.1016/j.ijrobp.2013.01.011 PMC3731128 23433795

[35] Chang HHY Pannunzio NR Adachi N Lieber MR. Non-homologous DNA end joining and alternative pathways to double-strand break repair. Nat Rev Mol Cell Biol. (2017) 18:495–506. doi: 10.1038/nrm.2017.48 28512351 PMC7062608

[36] Pannunzio NR Watanabe G Lieber MR. Nonhomologous DNA end-joining for repair of DNA double-strand breaks. J Biol Chem. (2018) 293:10512–23. doi: 10.1074/jbc.TM117.000374 PMC6036208 29247009

[37] Sishc BJ Davis AJ. The role of the core non-homologous end joining factors in carcinogenesis and cancer. Cancers (Basel). (2017) 9:81. doi: 10.3390/cancers9070081 28684677 PMC5532617

[38] Wright WD Shah SS Heyer WD. Homologous recombination and the repair of DNA double-strand breaks. J Biol Chem. (2018) 293:10524–35. doi: 10.1074/jbc.TM118.000372 PMC6036207 29599286

[39] Li X Heyer WD. Homologous recombination in DNA repair and DNA damage tolerance. Cell Res. (2008) 18:99–113. doi: 10.1038/cr.2008.1 18166982 PMC3087377

[40] Deans AJ West SC. DNA interstrand crosslink repair and cancer. Nat Rev Cancer. (2011) 11:467–80. doi: 10.1038/nrc3088 PMC3560328 21701511

[41] Hashimoto S Anai H Hanada K. Mechanisms of interstrand DNA crosslink repair and human disorders. Genes Environment. (2016) 38:9. doi: 10.1186/s41021-016-0037-9 27350828 PMC4918140

[42] Loeb LA Bielas JH Beckman RA. Cancers exhibit a mutator phenotype: clinical implications. Cancer Res. (2008) 68:3551–7. doi: 10.1158/0008-5472.CAN-07-5835 18483233

[43] Heinen CD Schmutte C Fishel R. DNA repair and tumorigenesis: lessons from hereditary cancer syndromes. Cancer Biol Ther. (2002) 1:477–85. doi: 10.4161/cbt.1.5.160 12496472

[44] Abdel-Fatah TM Russell R Agarwal D Moseley P Abayomi MA Perry C. DNA polymerase beta deficiency is linked to aggressive breast cancer: a comprehensive analysis of gene copy number, mRNA and protein expression in multiple cohorts. Mol Oncol. (2014) 8:520–32. doi: 10.1016/j.molonc.2014.01.001 PMC5528629 24462520

[45] Gachechiladze M Skarda J Bouchalova K Soltermann A Joerger M. Predictive and prognostic value of DNA damage response associated kinases in solid tumors. Front Oncol. (2020) 10:581217. doi: 10.3389/fonc.2020.581217 33224881 PMC7670868

[46] Takeshita T Asaoka M Katsuta E Photiadis SJ Narayanan S Yan L. High expression of polo-like kinase 1 is associated with TP53 inactivation, DNA repair deficiency, and worse prognosis in ER positive Her2 negative breast cancer. Am J Transl Res. (2019) 11:6507–21. PMC6834504 31737202

[47] Wynne P Newton C Ledermann JA Olaitan A Mould TA Hartley JA. Enhanced repair of DNA interstrand crosslinking in ovarian cancer cells from patients following treatment with platinum-based chemotherapy. Br J Cancer. (2007) 97:927–33. doi: 10.1038/sj.bjc.6603973 PMC2360410 17848946

[48] Havasi A Cainap SS Havasi AT Cainap C. Ovarian cancer-insights into platinum resistance and overcoming it. Medicina (Kaunas). (2023) 59:544. doi: 10.3390/medicina59030544 36984544 PMC10057458

[49] du Bois A Luck HJ Meier W Adams HP Mobus V Costa S. A randomized clinical trial of cisplatin/paclitaxel versus carboplatin/paclitaxel as first-line treatment of ovarian cancer. J Natl Cancer Inst. (2003) 95:1320–9. doi: 10.1093/jnci/djg036 12953086

[50] O'Neil NJ Bailey ML Hieter P. Synthetic lethality and cancer. Nat Rev Genet. (2017) 18:613–23. doi: 10.1038/nrg.2017.47 28649135

[51] Ray Chaudhuri A Nussenzweig A. The multifaceted roles of PARP1 in DNA repair and chromatin remodelling. Nat Rev Mol Cell Biol. (2017) 18:610–21. doi: 10.1038/nrm.2017.53 PMC6591728 28676700

[52] van Beek L McClay E Patel S Schimpl M Spagnolo L Maia de Oliveira T. PARP power: A structural perspective on PARP1, PARP2, and PARP3 in DNA damage repair and nucleosome remodelling. Int J Mol Sci. (2021) 22:5112. doi: 10.3390/ijms22105112 34066057 PMC8150716

[53] Barkauskaite E Jankevicius G Ahel I. Structures and mechanisms of enzymes employed in the synthesis and degradation of PARP-dependent protein ADP-ribosylation. Mol Cell. (2015) 58:935–46. doi: 10.1016/j.molcel.2015.05.007 26091342

[54] D'Amours D Desnoyers S D'Silva I Poirier GG. Poly(ADP-ribosyl)ation reactions in the regulation of nuclear functions. Biochem J. (1999) 342:249–68. doi: 10.1042/bj3420249 PMC1220459 10455009

[55] Krietsch J Rouleau M Pic E Ethier C Dawson TM Dawson VL. Reprogramming cellular events by poly(ADP-ribose)-binding proteins. Mol Aspects Med. (2013) 34:1066–87. doi: 10.1016/j.mam.2012.12.005 PMC3812366 23268355

[56] Javle M Curtin NJ. The role of PARP in DNA repair and its therapeutic exploitation. Br J Cancer. (2011) 105:1114–22. doi: 10.1038/bjc.2011.382 PMC3208503 21989215

[57] Langelier MF Riccio AA Pascal JM. PARP-2 and PARP-3 are selectively activated by 5' phosphorylated DNA breaks through an allosteric regulatory mechanism shared with PARP-1. Nucleic Acids Res. (2014) 42:7762–75. doi: 10.1093/nar/gku474 PMC4081085 24928857

[58] Rose M Burgess JT O'Byrne K Richard DJ Bolderson E. PARP inhibitors: clinical relevance, mechanisms of action and tumor resistance. Front Cell Dev Biol. (2020) 8:564601. doi: 10.3389/fcell.2020.564601 33015058 PMC7509090

[59] Marteijn JA Lans H Vermeulen W Hoeijmakers JH. Understanding nucleotide excision repair and its roles in cancer and ageing. Nat Rev Mol Cell Biol. (2014) 15:465–81. doi: 10.1038/nrm3822 24954209

[60] Haince JF McDonald D Rodrigue A Dery U Masson JY Hendzel MJ. PARP1-dependent kinetics of recruitment of MRE11 and NBS1 proteins to multiple DNA damage sites. J Biol Chem. (2008) 283:1197–208. doi: 10.1074/jbc.M706734200 18025084

[61] Ruscetti T Lehnert BE Halbrook J Le Trong H Hoekstra MF Chen DJ. Stimulation of the DNA-dependent protein kinase by poly(ADP-ribose) polymerase. J Biol Chem. (1998) 273:14461–7. doi: 10.1074/jbc.273.23.14461 9603959

[62] Rybanska I Ishaq O Chou J Prakash M Bakhsheshian J Huso DL. PARP1 and DNA-PKcs synergize to suppress p53 mutation and telomere fusions during T-lineage lymphomagenesis. Oncogene. (2013) 32:1761–71. doi: 10.1038/onc.2012.199 PMC4710090 22614020

[63] Li M Yu X. Function of BRCA1 in the DNA damage response is mediated by ADP-ribosylation. Cancer Cell. (2013) 23:693–704. doi: 10.1016/j.ccr.2013.03.025 23680151 PMC3759356

[64] Schwertman P Bekker-Jensen S Mailand N. Regulation of DNA double-strand break repair by ubiquitin and ubiquitin-like modifiers. Nat Rev Mol Cell Biol. (2016) 17:379–94. doi: 10.1038/nrm.2016.58 27211488

[65] Lau CH Seow KM Chen KH. The molecular mechanisms of actions, effects, and clinical implications of PARP inhibitors in epithelial ovarian cancers: A systematic review. Int J Mol Sci. (2022) 23:8125. doi: 10.3390/ijms23158125 35897700 PMC9332395

[66] Murai J Huang SY Das BB Renaud A Zhang Y Doroshow JH. Trapping of PARP1 and PARP2 by clinical PARP inhibitors. Cancer Res. (2012) 72:5588–99. doi: 10.1158/0008-5472.CAN-12-2753 PMC3528345 23118055

[67] Cong K Peng M Kousholt AN Lee WTC Lee S Nayak S. Replication gaps are a key determinant of PARP inhibitor synthetic lethality with BRCA deficiency. Mol Cell. (2021) 81:3128–44 e7. doi: 10.1016/j.molcel.2021.06.011 34216544 PMC9089372

[68] Lord CJ Ashworth A. BRCAness revisited. Nat Rev Cancer. (2016) 16:110–20. doi: 10.1038/nrc.2015.21 26775620

[69] Farmer H McCabe N Lord CJ Tutt AN Johnson DA Richardson TB. Targeting the DNA repair defect in BRCA mutant cells as a therapeutic strategy. Nature. (2005) 434:917–21. doi: 10.1038/nature03445 15829967

[70] Bryant HE Schultz N Thomas HD Parker KM Flower D Lopez E. Specific killing of BRCA2-deficient tumours with inhibitors of poly(ADP-ribose) polymerase. Nature. (2005) 434:913–7. doi: 10.1038/nature03443 15829966

[71] Moore K Colombo N Scambia G Kim BG Oaknin A Friedlander M. Maintenance olaparib in patients with newly diagnosed advanced ovarian cancer. N Engl J Med. (2018) 379:2495–505. doi: 10.1056/NEJMoa1810858 30345884

[72] Banerjee S Moore KN Colombo N Scambia G Kim BG Oaknin A. Maintenance olaparib for patients with newly diagnosed advanced ovarian cancer and a BRCA mutation (SOLO1/GOG 3004): 5-year follow-up of a randomised, double-blind, placebo-controlled, phase 3 trial. Lancet Oncol. (2021) 22:1721–31. doi: 10.1016/S1470-2045(21)00531-3 34715071

[73] DiSilvestro P Banerjee S Colombo N Scambia G Kim BG Oaknin A. Overall survival with maintenance olaparib at a 7-year follow-up in patients with newly diagnosed advanced ovarian cancer and a BRCA mutation: the SOLO1/GOG 3004 trial. J Clin Oncol. (2023) 41:609–17. doi: 10.1200/JCO.22.01549 PMC9870219 36082969

[74] Ray-Coquard I Pautier P Pignata S Perol D Gonzalez-Martin A Berger R. Olaparib plus bevacizumab as first-line maintenance in ovarian cancer. N Engl J Med. (2019) 381:2416–28. doi: 10.1056/NEJMoa1911361 31851799

[75] Pujade-Lauraine E Ledermann JA Selle F Gebski V Penson RT Oza AM. Olaparib tablets as maintenance therapy in patients with platinum-sensitive, relapsed ovarian cancer and a BRCA1/2 mutation (SOLO2/ENGOT-Ov21): a double-blind, randomised, placebo-controlled, phase 3 trial. Lancet Oncol. (2017) 18:1274–84. doi: 10.1016/S1470-2045(17)30469-2 28754483

[76] Chan N Koritzinsky M Zhao H Bindra R Glazer PM Powell S. Chronic hypoxia decreases synthesis of homologous recombination proteins to offset chemoresistance and radioresistance. Cancer Res. (2008) 68:605–14. doi: 10.1158/0008-5472.CAN-07-5472 18199558

[77] Kaplan AR Gueble SE Liu Y Oeck S Kim H Yun Z. Cediranib suppresses homology-directed DNA repair through down-regulation of BRCA1/2 and RAD51. Sci Transl Med. (2019) 11:eaav4508. doi: 10.1126/scitranslmed.aav4508 31092693 PMC6626544

[78] Sekine M Nishino K Enomoto T. BRCA genetic test and risk-reducing salpingo-oophorectomy for hereditary breast and ovarian cancer: state-of-the-art. Cancers (Basel). (2021) 13:2562. doi: 10.3390/cancers13112562 34071148 PMC8197088

[79] Ray-Coquard I Leary A Pignata S Cropet C Gonzalez-Martin A Marth C. Olaparib plus bevacizumab first-line maintenance in ovarian cancer: final overall survival results from the PAOLA-1/ENGOT-ov25 trial. Ann Oncol. (2023) 34:681–92. doi: 10.1016/j.annonc.2023.05.005 37211045

[80] Poveda A Floquet A Ledermann JA Asher R Penson RT Oza AM. Olaparib tablets as maintenance therapy in patients with platinum-sensitive relapsed ovarian cancer and a BRCA1/2 mutation (SOLO2/ENGOT-Ov21): a final analysis of a double-blind, randomised, placebo-controlled, phase 3 trial. Lancet Oncol. (2021) 22:620–31. doi: 10.1016/S1470-2045(21)00073-5 33743851

[81] Pujade-Lauraine E Selle F Scambia G Asselain B Marme F Lindemann K. Maintenance olaparib rechallenge in patients with platinum-sensitive relapsed ovarian cancer previously treated with a PARP inhibitor (OReO/ENGOT-ov38): a phase IIIb trial. Ann Oncol. (2023) 34:1152–64. doi: 10.1016/j.annonc.2023.09.3110 37797734

[82] Coleman RL Oza AM Lorusso D Aghajanian C Oaknin A Dean A. Rucaparib maintenance treatment for recurrent ovarian carcinoma after response to platinum therapy (ARIEL3): a randomised, double-blind, placebo-controlled, phase 3 trial. Lancet. (2017) 390:1949–61. doi: 10.1016/S0140-6736(17)32440-6 PMC5901715 28916367

[83] Coleman RL Oza A Lorusso D Aghajanian C Oaknin A Dean A. O003/557 Overall survival results from ARIEL3: a phase 3 randomized, double-blind study of rucaparib vs placebo following response to platinum-based chemotherapy for recurrent ovarian carcinoma. Int J Gynecologic Cancer. (2022) 32:A3–4. doi: 10.1136/ijgc-2022-igcs.5

[84] Kristeleit R Lisyanskaya A Fedenko A Dvorkin M de Melo AC Shparyk Y. Rucaparib versus standard-of-care chemotherapy in patients with relapsed ovarian cancer and a deleterious BRCA1 or BRCA2 mutation (ARIEL4): an international, open-label, randomised, phase 3 trial. Lancet Oncol. (2022) 23:465–78. doi: 10.1016/S1470-2045(22)00122-X 35298906

[85] Oza AM Lisyanskaya AS Fedenko AA de Melo AC Shparik Y Bondarenko I. 518O Overall survival results from ARIEL4: A phase III study assessing rucaparib vs chemotherapy in patients with advanced, relapsed ovarian carcinoma and a deleterious BRCA1/2 mutation. Ann Oncol. (2022) 33:S780. doi: 10.1016/j.annonc.2022.07.646

[86] Gonzalez-Martin A Pothuri B Vergote I DePont Christensen R Graybill W Mirza MR. Niraparib in patients with newly diagnosed advanced ovarian cancer. N Engl J Med. (2019) 381:2391–402. doi: 10.1056/NEJMoa1910962 31562799

[87] Gonzalez-Martin A Pothuri B Vergote I Graybill W Lorusso D McCormick CC. Progression-free survival and safety at 3.5years of follow-up: results from the randomised phase 3 PRIMA/ENGOT-OV26/GOG-3012 trial of niraparib maintenance treatment in patients with newly diagnosed ovarian cancer. Eur J Cancer. (2023) 189:112908. doi: 10.1016/j.ejca.2023.04.024 37263896

[88] Mirza MR Monk BJ Herrstedt J Oza AM Mahner S Redondo A. Niraparib maintenance therapy in platinum-sensitive, recurrent ovarian cancer. N Engl J Med. (2016) 375:2154–64. doi: 10.1056/NEJMoa1611310 27717299

[89] Mirza MR Herrstedt J Oza A Mahner S Redondo A Berton D. 161 Final overall survival and long-term safety in the ENGOT-OV16/NOVA phase 3 trial of niraparib in patients with recurrent ovarian cancer. Int J Gynecologic Cancer. (2023) 33:A15–A6. doi: 10.1136/ijgc-2023-ESGO.22

[90] Shen Y Rehman FL Feng Y Boshuizen J Bajrami I Elliott R. BMN 673, a novel and highly potent PARP1/2 inhibitor for the treatment of human cancers with DNA repair deficiency. Clin Cancer Res. (2013) 19:5003–15. doi: 10.1158/1078-0432.CCR-13-1391 PMC6485449 23881923

[91] Bao S Yue Y Hua Y Zeng T Yang Y Yang F. Safety profile of poly (ADP-ribose) polymerase (PARP) inhibitors in cancer: a network meta-analysis of randomized controlled trials. Ann Trans Med. (2021) 9:1229. doi: 10.21037/atm PMC8421942 34532366

[92] de Bono J Ramanathan RK Mina L Chugh R Glaspy J Rafii S. Dose-escalation, two-part trial of the PARP inhibitor talazoparib in patients with advanced germline BRCA1/2 mutations and selected sporadic cancers. Cancer Discovery. (2017) 7:620–9. doi: 10.1158/2159-8290.CD-16-1250 PMC5905335 28242752

[93] Dhawan MS Bartelink IH Aggarwal RR Leng J Zhang JZ Pawlowska N. Differential toxicity in patients with and without DNA repair mutations: phase I study of carboplatin and talazoparib in advanced solid tumors. Clin Cancer Res. (2017) 23:6400–10. doi: 10.1158/1078-0432.CCR-17-0703 28790114

[94] Boussios S Abson C Moschetta M Rassy E Karathanasi A Bhat T. Poly (ADP-ribose) polymerase inhibitors: talazoparib in ovarian cancer and beyond. Drugs R D. (2020) 20:55–73. doi: 10.1007/s40268-020-00301-8 32215876 PMC7221042

[95] Coleman RL Fleming GF Brady MF Swisher EM Steffensen KD Friedlander M. Veliparib with first-line chemotherapy and as maintenance therapy in ovarian cancer. N Engl J Med. (2019) 381:2403–15. doi: 10.1056/NEJMoa1909707 PMC6941439 31562800

[96] Swisher EM Aghajanian C O'Malley DM Fleming GF Kaufmann SH Levine DA. Impact of homologous recombination status and responses with veliparib combined with first-line chemotherapy in ovarian cancer in the Phase 3 VELIA/GOG-3005 study. Gynecol Oncol. (2022) 164:245–53. doi: 10.1016/j.ygyno.2021.12.003 34906376

[97] Lee A. Fuzuloparib: first approval. Drugs. (2021) 81:1221–6. doi: 10.1007/s40265-021-01541-x PMC8380563 34118019

[98] Li N Liu Q Tian Y Wu L. Overview of fuzuloparib in the treatment of ovarian cancer: background and future perspective. J Gynecol Oncol. (2022) 33:e86. doi: 10.3802/jgo.2022.33.e86 36335989 PMC9634097

[99] Li N Bu H Liu J Zhu J Zhou Q Wang L. An open-label, multicenter, single-arm, phase II study of fluzoparib in patients with germline BRCA1/2 mutation and platinum-sensitive recurrent ovarian cancer. Clin Cancer Res. (2021) 27:2452–8. doi: 10.1158/1078-0432.CCR-20-3546 33558426

[100] Friedlander M Mileshkin L Lombard J Frentzas S Gao B Wilson M. Pamiparib in combination with tislelizumab in patients with advanced solid tumours: results from the dose-expansion stage of a multicentre, open-label, phase I trial. Br J Cancer. (2023) 129:797–810. doi: 10.1038/s41416-023-02349-0 37474720 PMC10449784

[101] Wu X Zhu J Wang J Lin Z Yin R Sun W. Pamiparib monotherapy for patients with germline BRCA1/2-mutated ovarian cancer previously treated with at least two lines of chemotherapy: A multicenter, open-label, phase II study. Clin Cancer Res. (2022) 28:653–61. doi: 10.1158/1078-0432.CCR-21-1186 PMC9377729 34844979

[102] LaFargue CJ Dal Molin GZ Sood AK Coleman RL. Exploring and comparing adverse events between PARP inhibitors. Lancet Oncol. (2019) 20:e15–28. doi: 10.1016/S1470-2045(18)30786-1 PMC7292736 30614472

[103] Friedlander M Lee YC Tew WP. Managing adverse effects associated with poly (ADP-ribose) polymerase inhibitors in ovarian cancer: A synthesis of clinical trial and real-world data. Am Soc Clin Oncol Educ Book. (2023) 43):e390876. doi: 10.1200/EDBK_390876 37285556

[104] Farres J Llacuna L Martin-Caballero J Martinez C Lozano JJ Ampurdanes C. PARP-2 sustains erythropoiesis in mice by limiting replicative stress in erythroid progenitors. Cell Death Differ. (2015) 22:1144–57. doi: 10.1038/cdd.2014.202 PMC4568570 25501596

[105] Oza AM Cibula D Benzaquen AO Poole C Mathijssen RH Sonke GS. Olaparib combined with chemotherapy for recurrent platinum-sensitive ovarian cancer: a randomised phase 2 trial. Lancet Oncol. (2015) 16:87–97. doi: 10.1016/S1470-2045(14)71135-0 25481791

[106] Yamaoka K Fujiwara M Uchida M Uesawa Y Muroi N Shimizu T. Comprehensive analysis of adverse events induced by PARP inhibitors using JADER and time to onset. Life (Basel). (2022) 12:1355. doi: 10.3390/life12091355 36143391 PMC9504973

[107] Mateo J Lord CJ Serra V Tutt A Balmana J Castroviejo-Bermejo M. A decade of clinical development of PARP inhibitors in perspective. Ann Oncol. (2019) 30:1437–47. doi: 10.1093/annonc/mdz192 PMC6771225 31218365

[108] Lord CJ Ashworth A. PARP inhibitors: Synthetic lethality in the clinic. Science. (2017) 355:1152–8. doi: 10.1126/science.aam7344 PMC6175050 28302823

[109] Valabrega G Scotto G Tuninetti V Pani A Scaglione F. Differences in PARP inhibitors for the treatment of ovarian cancer: mechanisms of action, pharmacology, safety, and efficacy. Int J Mol Sci. (2021) 22:4203. doi: 10.3390/ijms22084203 33921561 PMC8073512

[110] Chan JK Liu J Song J Xiang C Wu E Kalilani L. Real-world outcomes associated with poly(ADP-ribose) polymerase inhibitor monotherapy maintenance in patients with primary advanced ovarian cancer. Am J Clin Oncol. (2023) 46:314–22. doi: 10.1097/COC.0000000000001010 PMC10281176 37106485

[111] Reid RL Shi J Monnette A Wallace KL. Real-world progression-free and overall survival for patients with advanced ovarian cancer utilizing PARP inhibitor second-line maintenance therapy vs active surveillance. J Clin Oncol. (2022) 40:e18812–e. doi: 10.1200/JCO.2022.40.16_suppl.e18812

[112] Vilming B Fallas Dahl J Bentzen AG Ingebrigtsen VA Berge Nilsen E Vistad I. Real-world data on niraparib maintenance treatment in patients with non-gBRCA mutated platinum-sensitive recurrent ovarian cancer. Int J Gynecol Cancer. (2023) 33:1898–905. doi: 10.1136/ijgc-2023-004484 PMC10803952 38000795

[113] Pan YE Hood A Ahmad H Altwerger G. Real-world efficacy and safety of PARP inhibitors in recurrent ovarian cancer patients with somatic BRCA and other homologous recombination gene mutations. Ann Pharmacother. (2023) 57:1162–71. doi: 10.1177/10600280221149136 PMC11062080 36651235

[114] Lheureux S Oaknin A Garg S Bruce JP Madariaga A Dhani NC. EVOLVE: A multicenter open-label single-arm clinical and translational phase II trial of cediranib plus olaparib for ovarian cancer after PARP inhibition progression. Clin Cancer Res. (2020) 26:4206–15. doi: 10.1158/1078-0432.CCR-19-4121 32444417

[115] Tobalina L Armenia J Irving E O'Connor MJ Forment JV. A meta-analysis of reversion mutations in BRCA genes identifies signatures of DNA end-joining repair mechanisms driving therapy resistance. Ann Oncol. (2021) 32:103–12. doi: 10.1016/j.annonc.2020.10.470 33091561

[116] Drean A Williamson CT Brough R Brandsma I Menon M Konde A. Modeling therapy resistance in BRCA1/2-mutant cancers. Mol Cancer Ther. (2017) 16:2022–34. doi: 10.1158/1535-7163.MCT-17-0098 PMC6157714 28619759

[117] Wang N Yang Y Jin D Zhang Z Shen K Yang J. PARP inhibitor resistance in breast and gynecological cancer: Resistance mechanisms and combination therapy strategies. Front Pharmacol. (2022) 13:967633. doi: 10.3389/fphar.2022.967633 36091750 PMC9455597

[118] Ter Brugge P Kristel P van der Burg E Boon U de Maaker M Lips E. Mechanisms of therapy resistance in patient-derived xenograft models of BRCA1-deficient breast cancer. J Natl Cancer Inst. (2016) 108. doi: 10.1093/jnci/djw148 27381626

[119] Kondrashova O Topp M Nesic K Lieschke E Ho GY Harrell MI. Methylation of all BRCA1 copies predicts response to the PARP inhibitor rucaparib in ovarian carcinoma. Nat Commun. (2018) 9:3970. doi: 10.1038/s41467-018-05564-z 30266954 PMC6162272

[120] Jackson LM Moldovan GL. Mechanisms of PARP1 inhibitor resistance and their implications for cancer treatment. NAR Cancer. (2022) 4:zcac042. doi: 10.1093/narcan/zcac042 36568963 PMC9773381

[121] Hashimoto Y Ray Chaudhuri A Lopes M Costanzo V. Rad51 protects nascent DNA from Mre11-dependent degradation and promotes continuous DNA synthesis. Nat Struct Mol Biol. (2010) 17:1305–11. doi: 10.1038/nsmb.1927 PMC4306207 20935632

[122] Guffanti F Alvisi MF Anastasia A Ricci F Chiappa M Llop-Guevara A. Basal expression of RAD51 foci predicts olaparib response in patient-derived ovarian cancer xenografts. Br J Cancer. (2022) 126:120–8. doi: 10.1038/s41416-021-01609-1 PMC8727677 34732853

[123] Castroviejo-Bermejo M Cruz C Llop-Guevara A Gutierrez-Enriquez S Ducy M Ibrahim YH. A RAD51 assay feasible in routine tumor samples calls PARP inhibitor response beyond BRCA mutation. EMBO Mol Med. (2018) 10:e9172. doi: 10.15252/emmm.201809172 30377213 PMC6284440

[124] Cruz C Castroviejo-Bermejo M Gutierrez-Enriquez S Llop-Guevara A Ibrahim YH Gris-Oliver A. RAD51 foci as a functional biomarker of homologous recombination repair and PARP inhibitor resistance in germline BRCA-mutated breast cancer. Ann Oncol. (2018) 29:1203–10. doi: 10.1093/annonc/mdy099 PMC5961353 29635390

[125] Kondrashova O Nguyen M Shield-Artin K Tinker AV Teng NNH Harrell MI. Secondary somatic mutations restoring RAD51C and RAD51D associated with acquired resistance to the PARP inhibitor rucaparib in high-grade ovarian carcinoma. Cancer Discovery. (2017) 7:984–98. doi: 10.1158/2159-8290.CD-17-0419 PMC5612362 28588062

[126] Nesic K Kondrashova O Hurley RM McGehee CD Vandenberg CJ Ho GY. Acquired RAD51C promoter methylation loss causes PARP inhibitor resistance in high-grade serous ovarian carcinoma. Cancer Res. (2021) 81:4709–22. doi: 10.1158/0008-5472.CAN-21-0774 PMC12593227 34321239

[127] van Wijk LM Nilas AB Vrieling H Vreeswijk MPG. RAD51 as a functional biomarker for homologous recombination deficiency in cancer: a promising addition to the HRD toolbox? Expert Rev Mol Diagn. (2022) 22:185–99. doi: 10.1080/14737159.2022.2020102 34913794

[128] Kim D Nam HJ. PARP inhibitors: clinical limitations and recent attempts to overcome them. Int J Mol Sci. (2022) 23:8142. doi: 10.3390/ijms23158412 35955544 PMC9369301

[129] Xu G Chapman JR Brandsma I Yuan J Mistrik M Bouwman P. REV7 counteracts DNA double-strand break resection and affects PARP inhibition. Nature. (2015) 521:541–4. doi: 10.1038/nature14328 PMC4671316 25799992

[130] Hurley RM Wahner Hendrickson AE Visscher DW Ansell P Harrell MI Wagner JM. 53BP1 as a potential predictor of response in PARP inhibitor-treated homologous recombination-deficient ovarian cancer. Gynecol Oncol. (2019) 153:127–34. doi: 10.1016/j.ygyno.2019.01.015 PMC6430710 30686551

[131] Bouwman P Aly A Escandell JM Pieterse M Bartkova J van der Gulden H. 53BP1 loss rescues BRCA1 deficiency and is associated with triple-negative and BRCA-mutated breast cancers. Nat Struct Mol Biol. (2010) 17:688–95. doi: 10.1038/nsmb.1831 PMC2912507 20453858

[132] Dev H Chiang TW Lescale C de Krijger I Martin AG Pilger D. Shieldin complex promotes DNA end-joining and counters homologous recombination in BRCA1-null cells. Nat Cell Biol. (2018) 20:954–65. doi: 10.1038/s41556-018-0140-1 PMC6145444 30022119

[133] Noordermeer SM Adam S Setiaputra D Barazas M Pettitt SJ Ling AK. The shieldin complex mediates 53BP1-dependent DNA repair. Nature. (2018) 560:117–21. doi: 10.1038/s41586-018-0340-7 PMC6141009 30022168

[134] Yang ZM Liao XM Chen Y Shen YY Yang XY Su Y. Combining 53BP1 with BRCA1 as a biomarker to predict the sensitivity of poly(ADP-ribose) polymerase (PARP) inhibitors. Acta Pharmacol Sin. (2017) 38:1038–47. doi: 10.1038/aps.2017.8 PMC5519258 28414200

[135] Gorski JW Ueland FR Kolesar JM. CCNE1 amplification as a predictive biomarker of chemotherapy resistance in epithelial ovarian cancer. Diagnostics (Basel). (2020) 10:279. doi: 10.3390/diagnostics10050279 32380689 PMC7277958

[136] Mei L Zhang J He K Zhang J. Ataxia telangiectasia and Rad3-related inhibitors and cancer therapy: where we stand. J Hematol Oncol. (2019) 12:43. doi: 10.1186/s13045-019-0733-6 31018854 PMC6482552

[137] Katayama K Fujita N Tsuruo T. Akt/protein kinase B-dependent phosphorylation and inactivation of WEE1Hu promote cell cycle progression at G2/M transition. Mol Cell Biol. (2005) 25:5725–37. doi: 10.1128/MCB.25.13.5725-5737.2005 PMC1156994 15964826

[138] Kim H George E Ragland R Rafail S Zhang R Krepler C. Targeting the ATR/CHK1 axis with PARP inhibition results in tumor regression in BRCA-mutant ovarian cancer models. Clin Cancer Res. (2017) 23:3097–108. doi: 10.1158/1078-0432.CCR-16-2273 PMC5474193 27993965

[139] Kim H Xu H George E Hallberg D Kumar S Jagannathan V. Combining PARP with ATR inhibition overcomes PARP inhibitor and platinum resistance in ovarian cancer models. Nat Commun. (2020) 11:3726. doi: 10.1038/s41467-020-17127-2 32709856 PMC7381609

[140] Coleman N Zhang B Byers LA Yap TA. The role of Schlafen 11 (SLFN11) as a predictive biomarker for targeting the DNA damage response. Br J Cancer. (2021) 124:857–9. doi: 10.1038/s41416-020-01202-y PMC7921443 33328609

[141] Murai J Feng Y Yu GK Ru Y Tang SW Shen Y. Resistance to PARP inhibitors by SLFN11 inactivation can be overcome by ATR inhibition. Oncotarget. (2016) 7:76534–50. doi: 10.18632/oncotarget.v7i47 PMC5340226 27708213

[142] Lok BH Gardner EE Schneeberger VE Ni A Desmeules P Rekhtman N. PARP inhibitor activity correlates with SLFN11 expression and demonstrates synergy with temozolomide in small cell lung cancer. Clin Cancer Res. (2017) 23:523–35. doi: 10.1158/1078-0432.CCR-16-1040 PMC5241177 27440269

[143] Allison Stewart C Tong P Cardnell RJ Sen T Li L Gay CM. Dynamic variations in epithelial-to-mesenchymal transition (EMT), ATM, and SLFN11 govern response to PARP inhibitors and cisplatin in small cell lung cancer. Oncotarget. (2017) 8:28575–87. doi: 10.18632/oncotarget.v8i17 PMC5438673 28212573

[144] van Erp AEM van Houdt L Hillebrandt-Roeffen MHS van Bree N Flucke UE Mentzel T. Olaparib and temozolomide in desmoplastic small round cell tumors: a promising combination in *vitro* and in *vivo* . J Cancer Res Clin Oncol. (2020) 146:1659–70. doi: 10.1007/s00432-020-03211-z PMC7256072 32279088

[145] Pietanza MC Waqar SN Krug LM Dowlati A Hann CL Chiappori A. Randomized, double-blind, phase II study of temozolomide in combination with either veliparib or placebo in patients with relapsed-sensitive or refractory small-cell lung cancer. J Clin Oncol. (2018) 36:2386–94. doi: 10.1200/JCO.2018.77.7672 PMC6085179 29906251

[146] Karim NFA Miao J Reckamp KL Gay CM Byers LA Zhao Y. SWOG S1929: Phase II randomized study of maintenance atezolizumab (A) versus atezolizumab + talazoparib (AT) in patients with SLFN11 positive extensive stage small cell lung cancer (ES-SCLC). J Clin Oncol. (2023) 41:8504. doi: 10.1200/JCO.2023.41.16_suppl.8504 PMC13409069 39505259

[147] Zatreanu D Robinson HMR Alkhatib O Boursier M Finch H Geo L. Poltheta inhibitors elicit BRCA-gene synthetic lethality and target PARP inhibitor resistance. Nat Commun. (2021) 12:3636. doi: 10.1038/s41467-021-23463-8 34140467 PMC8211653

[148] Gogola E Duarte AA de Ruiter JR Wiegant WW Schmid JA de Bruijn R. Selective loss of PARG restores PARylation and counteracts PARP inhibitor-mediated synthetic lethality. Cancer Cell. (2018) 33:1078–93 e12. doi: 10.1016/j.ccell.2018.05.008 29894693

[149] Min W Bruhn C Grigaravicius P Zhou ZW Li F Kruger A. Poly(ADP-ribose) binding to Chk1 at stalled replication forks is required for S-phase checkpoint activation. Nat Commun. (2013) 4:2993. doi: 10.1038/ncomms3993 24356582

[150] Pillay N Tighe A Nelson L Littler S Coulson-Gilmer C Bah N. DNA replication vulnerabilities render ovarian cancer cells sensitive to poly(ADP-ribose) glycohydrolase inhibitors. Cancer Cell. (2019) 35:519–33 e8. doi: 10.1016/j.ccell.2019.02.004 30889383 PMC6428690

[151] Pettitt SJ Krastev DB Brandsma I Drean A Song F Aleksandrov R. Genome-wide and high-density CRISPR-Cas9 screens identify point mutations in PARP1 causing PARP inhibitor resistance. Nat Commun. (2018) 9:1849. doi: 10.1038/s41467-018-03917-2 29748565 PMC5945626

[152] Veneziani AC Scott C Wakefield MJ Tinker AV Lheureux S. Fighting resistance: post-PARP inhibitor treatment strategies in ovarian cancer. Ther Adv Med Oncol. (2023) 15:17588359231157644. doi: 10.1177/17588359231157644 36872947 PMC9983116

[153] Christie EL Pattnaik S Beach J Copeland A Rashoo N Fereday S. Multiple ABCB1 transcriptional fusions in drug resistant high-grade serous ovarian and breast cancer. Nat Commun. (2019) 10:1295. doi: 10.1038/s41467-019-09312-9 30894541 PMC6426934

[154] Vaidyanathan A Sawers L Gannon AL Chakravarty P Scott AL Bray SE. ABCB1 (MDR1) induction defines a common resistance mechanism in paclitaxel- and olaparib-resistant ovarian cancer cells. Br J Cancer. (2016) 115:431–41. doi: 10.1038/bjc.2016.203 PMC4985349 27415012

[155] Lawlor D Martin P Busschots S Thery J O'Leary JJ Hennessy BT. PARP inhibitors as P-glyoprotein substrates. J Pharm Sci. (2014) 103:1913–20. doi: 10.1002/jps.23952 24700236

[156] Deng M Qin K Li Y Jiang Y Jin W Shen Z. Abstract 5338: Pamiparib as a non-P-glycoprotein substrate PARP inhibitor can overcome ABCB1-mediated multidrug resistance in ovarian cancer cells. Cancer Res. (2022) 82:5338. doi: 10.1158/1538-7445.AM2022-5338

[157] Tunnage I Lara O Misirlioglu S Pereira LD Abdelrahman S Levine D. PARP and ABCB1 (MDR1) inhibitor treatment of ovarian cancer cell lines and PDX models demonstrate no synergistic effect (244). Gynecologic Oncol. (2022) 166:S132–S3. doi: 10.1016/S0090-8258(22)01467-6

[158] Ray Chaudhuri A Callen E Ding X Gogola E Duarte AA Lee JE. Replication fork stability confers chemoresistance in BRCA-deficient cells. Nature. (2016) 535:382–7. doi: 10.1038/nature18325 PMC4959813 27443740

[159] Taglialatela A Alvarez S Leuzzi G Sannino V Ranjha L Huang JW. Restoration of replication fork stability in BRCA1- and BRCA2-deficient cells by inactivation of SNF2-family fork remodelers. Mol Cell. (2017) 68:414–30 e8. doi: 10.1016/j.molcel.2017.09.036 29053959 PMC5720682

[160] Dungrawala H Bhat KP Le Meur R Chazin WJ Ding X Sharan SK. RADX promotes genome stability and modulates chemosensitivity by regulating RAD51 at replication forks. Mol Cell. (2017) 67:374–86 e5. doi: 10.1016/j.molcel.2017.06.023 28735897 PMC5548441

[161] Burdett NL Willis MO Pandey A Fereday S Group AS DeFazio A. Small-scale mutations are infrequent as mechanisms of resistance in post-PARP inhibitor tumour samples in high grade serous ovarian cancer. Sci Rep. (2023) 13:21884. doi: 10.1038/s41598-023-48153-x 38072854 PMC10711013

[162] Dockery LE Rubenstein AR Ding K Mashburn SG Burkett WC Davis AM. Extending the platinum-free interval: The impact of omitting 2nd line platinum chemotherapy in intermediate platinum-sensitive ovarian cancer. Gynecol Oncol. (2019) 155:201–6. doi: 10.1016/j.ygyno.2019.07.008 PMC7081661 31522837

[163] Stuart GC Kitchener H Bacon M duBois A Friedlander M Ledermann J. 2010 Gynecologic Cancer InterGroup (GCIG) consensus statement on clinical trials in ovarian cancer: report from the Fourth Ovarian Cancer Consensus Conference. Int J Gynecol Cancer. (2011) 21:750–5. doi: 10.1097/IGC.0b013e31821b2568 21543936

[164] Lindemann K Gao B Mapagu C Fereday S Emmanuel C Alsop K. Response rates to second-line platinum-based therapy in ovarian cancer patients challenge the clinical definition of platinum resistance. Gynecol Oncol. (2018) 150:239–46. doi: 10.1016/j.ygyno.2018.05.020 29807697

[165] Caruso G Tomao F Parma G Lapresa M Multinu F Palaia I. Poly (ADP-ribose) polymerase inhibitors (PARPi) in ovarian cancer: lessons learned and future directions. Int J Gynecol Cancer. (2023) 33:431–43. doi: 10.1136/ijgc-2022-004149 36928097

[166] Rocha CRR Silva MM Quinet A Cabral-Neto JB Menck CFM. DNA repair pathways and cisplatin resistance: an intimate relationship. Clinics (Sao Paulo). (2018) 73:e478s. doi: 10.6061/clinics/2018/e478s 30208165 PMC6113849

[167] Mylavarapu S Das A Roy M. Role of BRCA mutations in the modulation of response to platinum therapy. Front Oncol. (2018) 8:16. doi: 10.3389/fonc.2018.00016 29459887 PMC5807680

[168] Wen H Feng Z Ma Y Liu R Ou Q Guo Q. Homologous recombination deficiency in diverse cancer types and its correlation with platinum chemotherapy efficiency in ovarian cancer. BMC Cancer. (2022) 22:550. doi: 10.1186/s12885-022-09602-4 35578198 PMC9109318

[169] McMullen M Karakasis K Madariaga A Oza AM. Overcoming platinum and PARP-inhibitor resistance in ovarian cancer. Cancers (Basel). (2020) 12:1607. doi: 10.3390/cancers12061607 32560564 PMC7352566

[170] Huang D Savage SR Calinawan AP Lin C Zhang B Wang P. A highly annotated database of genes associated with platinum resistance in cancer. Oncogene. (2021) 40:6395–405. doi: 10.1038/s41388-021-02055-2 PMC8602037 34645978

[171] Maloney SM Hoover CA Morejon-Lasso LV Prosperi JR. Mechanisms of taxane resistance. Cancers (Basel). (2020) 12:3323. doi: 10.3390/cancers12113323 33182737 PMC7697134

[172] Frenel JS Kim JW Aryal N Asher R Berton D Vidal L. Efficacy of subsequent chemotherapy for patients with BRCA1/2-mutated recurrent epithelial ovarian cancer progressing on olaparib versus placebo maintenance: post-hoc analyses of the SOLO2/ENGOT Ov-21 trial. Ann Oncol. (2022) 33:1021–8. doi: 10.1016/j.annonc.2022.06.011 35772665

[173] Cecere SC Giannone G Salutari V Arenare L Lorusso D Ronzino G. Olaparib as maintenance therapy in patients with BRCA 1-2 mutated recurrent platinum sensitive ovarian cancer: Real world data and post progression outcome. Gynecol Oncol. (2020) 156:38–44. doi: 10.1016/j.ygyno.2019.10.023 31699415

[174] Romeo M Gil-Martin M Gaba L Teruel I Taus A Fina C. Multicenter real-world data of subsequent chemotherapy after progression to PARP inhibitors in a maintenance relapse setting. Cancers (Basel). (2022) 14:4414. doi: 10.3390/cancers14184414 36139574 PMC9497128

[175] Laine A Sims TT Le Saux O Ray-Coquard I Coleman RL. Treatment perspectives for ovarian cancer in Europe and the United States: initial therapy and platinum-sensitive recurrence after PARP inhibitors or bevacizumab therapy. Curr Oncol Rep. (2021) 23:148. doi: 10.1007/s11912-021-01128-5 34751835

[176] Aghajanian C Blank SV Goff BA Judson PL Teneriello MG Husain A. OCEANS: a randomized, double-blind, placebo-controlled phase III trial of chemotherapy with or without bevacizumab in patients with platinum-sensitive recurrent epithelial ovarian, primary peritoneal, or fallopian tube cancer. J Clin Oncol. (2012) 30:2039–45. doi: 10.1200/JCO.2012.42.0505 PMC3646321 22529265

[177] Coleman RL Brady MF Herzog TJ Sabbatini P Armstrong DK Walker JL. Bevacizumab and paclitaxel-carboplatin chemotherapy and secondary cytoreduction in recurrent, platinum-sensitive ovarian cancer (NRG Oncology/Gynecologic Oncology Group study GOG-0213): a multicentre, open-label, randomised, phase 3 trial. Lancet Oncol. (2017) 18:779–91. doi: 10.1016/S1470-2045(17)30279-6 PMC5715461 28438473

[178] Pignata S Lorusso D Joly F Gallo C Colombo N Sessa C. Carboplatin-based doublet plus bevacizumab beyond progression versus carboplatin-based doublet alone in patients with platinum-sensitive ovarian cancer: a randomised, phase 3 trial. Lancet Oncol. (2021) 22:267–76. doi: 10.1016/S1470-2045(20)30637-9 33539744

[179] Pfisterer J Shannon CM Baumann K Rau J Harter P Joly F. Bevacizumab and platinum-based combinations for recurrent ovarian cancer: a randomised, open-label, phase 3 trial. Lancet Oncol. (2020) 21:699–709. doi: 10.1016/S1470-2045(20)30142-X 32305099

[180] Pignata S Scambia G Bologna A Signoriello S Vergote IB Wagner U. Randomized controlled trial testing the efficacy of platinum-free interval prolongation in advanced ovarian cancer: the MITO-8, maNGO, BGOG-ov1, AGO-ovar2.16, ENGOT-ov1, GCIG study. J Clin Oncol. (2017) 35:3347–53. doi: 10.1200/JCO.2017.73.4293 28825853

[181] St Laurent J Liu JF. Treatment approaches for platinum-resistant ovarian cancer. J Clin Oncol. (2024) 42:127–33. doi: 10.1200/JCO.23.01771 37910841

[182] Pujade-Lauraine E Hilpert F Weber B Reuss A Poveda A Kristensen G. Bevacizumab combined with chemotherapy for platinum-resistant recurrent ovarian cancer: The AURELIA open-label randomized phase III trial. J Clin Oncol. (2014) 32:1302–8. doi: 10.1200/JCO.2013.51.4489 24637997

[183] Kim NK Kim Y Kim HS Park SJ Hwang DW Lee SJ. Risk factors for the failure of first-line PARP inhibitor maintenance therapy in patients with advanced ovarian cancer: Gynecologic Oncology Research Investigators Collaboration Study (GORILLA-3004). Cancer Med. (2023) 12:19449–59. doi: 10.1002/cam4.6546 PMC10587974 37768030

[184] Audeh MW Carmichael J Penson RT Friedlander M Powell B Bell-McGuinn KM. Oral poly(ADP-ribose) polymerase inhibitor olaparib in patients with BRCA1 or BRCA2 mutations and recurrent ovarian cancer: a proof-of-concept trial. Lancet. (2010) 376:245–51. doi: 10.1016/S0140-6736(10)60893-8 20609468

[185] Gelmon KA Tischkowitz M Mackay H Swenerton K Robidoux A Tonkin K. Olaparib in patients with recurrent high-grade serous or poorly differentiated ovarian carcinoma or triple-negative breast cancer: a phase 2, multicentre, open-label, non-randomised study. Lancet Oncol. (2011) 12:852–61. doi: 10.1016/S1470-2045(11)70214-5 21862407

[186] Jiang X Li X Li W Bai H Zhang Z. PARP inhibitors in ovarian cancer: Sensitivity prediction and resistance mechanisms. J Cell Mol Med. (2019) 23:2303–13. doi: 10.1111/jcmm.14133 PMC6433712 30672100

[187] Ewing A Meynert A Churchman M Grimes GR Hollis RL Herrington CS. Structural variants at the BRCA1/2 loci are a common source of homologous repair deficiency in high-grade serous ovarian carcinoma. Clin Cancer Res. (2021) 27:3201–14. doi: 10.1158/1078-0432.CCR-20-4068 PMC7610896 33741650

[188] Funingana IG Reinius MAV Petrillo A Ang JE Brenton JD. Can integrative biomarker approaches improve prediction of platinum and PARP inhibitor response in ovarian cancer? Semin Cancer Biol. (2021) 77:67–82. doi: 10.1016/j.semcancer.2021.02.008 33607245

[189] Telli ML Timms KM Reid J Hennessy B Mills GB Jensen KC. Homologous recombination deficiency (HRD) score predicts response to platinum-containing neoadjuvant chemotherapy in patients with triple-negative breast cancer. Clin Cancer Res. (2016) 22:3764–73. doi: 10.1158/1078-0432.CCR-15-2477 PMC6773427 26957554

[190] Skelin M Sarcevic D Lesin Gacina D Mucalo I Dilber I Javor E. The effect of PARP inhibitors in homologous recombination proficient ovarian cancer: meta-analysis. J Chemother. (2023) 35:150–7. doi: 10.1080/1120009X.2022.2073161 35550005

[191] Purwar R Ranjan R Pal M Upadhyay SK Kumar T Pandey M. Role of PARP inhibitors beyond BRCA mutation and platinum sensitivity in epithelial ovarian cancer: a meta-analysis of hazard ratios from randomized clinical trials. World J Surg Oncol. (2023) 21:157. doi: 10.1186/s12957-023-03027-4 37217940 PMC10204292

[192] Lee EK Konstantinopoulos PA. PARP inhibition and immune modulation: scientific rationale and perspectives for the treatment of gynecologic cancers. Ther Adv Med Oncol. (2020) 12:1758835920944116. doi: 10.1177/1758835920944116 32782491 PMC7383615

[193] Lin KK Harrell MI Oza AM Oaknin A Ray-Coquard I Tinker AV. BRCA reversion mutations in circulating tumor DNA predict primary and acquired resistance to the PARP inhibitor rucaparib in high-grade ovarian carcinoma. Cancer Discovery. (2019) 9:210–9. doi: 10.1158/2159-8290.CD-18-0715 30425037

[194] Lheureux S Prokopec SD Oldfield LE Gonzalez-Ochoa E Bruce JP Wong D. Identifying mechanisms of resistance by circulating tumor DNA in EVOLVE, a phase II trial of cediranib plus olaparib for ovarian cancer at time of PARP inhibitor progression. Clin Cancer Res. (2023) 29:3706–16. doi: 10.1158/1078-0432.CCR-23-0797 PMC10502468 37327320

[195] Sauer CM Heider K Belic J Boyle SE Hall JA Couturier DL. Longitudinal monitoring of disease burden and response using ctDNA from dried blood spots in xenograft models. EMBO Mol Med. (2022) 14:e15729. doi: 10.15252/emmm.202215729 35694774 PMC9358392

[196] Heider K Wan JCM Hall J Belic J Boyle S Hudecova I. Detection of ctDNA from Dried Blood Spots after DNA Size Selection. Clin Chem. (2020) 66:697–705. doi: 10.1093/clinchem/hvaa050 32268361

[197] Arcieri M Tius V Andreetta C Restaino S Biasioli A Poletto E. How BRCA and homologous recombination deficiency change therapeutic strategies in ovarian cancer: a review of literature. Front Oncol. (2024) 14:1335196. doi: 10.3389/fonc.2024.1335196 38525421 PMC10957789

[198] Pellegrino B Herencia-Ropero A Llop-Guevara A Pedretti F Moles-Fernandez A Viaplana C. Preclinical *in vivo* validation of the RAD51 test for identification of homologous recombination-deficient tumors and patient stratification. Cancer Res. (2022) 82:1646–57. doi: 10.1158/0008-5472.CAN-21-2409 PMC7612637 35425960

[199] Miller RE Elyashiv O El-Shakankery KH Ledermann JA. Ovarian cancer therapy: homologous recombination deficiency as a predictive biomarker of response to PARP inhibitors. Onco Targets Ther. (2022) 15:1105–17. doi: 10.2147/OTT.S272199 PMC9547601 36217436

[200] Makvandi M Pantel A Schwartz L Schubert E Xu K Hsieh CJ. A PET imaging agent for evaluating PARP-1 expression in ovarian cancer. J Clin Invest. (2018) 128:2116–26. doi: 10.1172/JCI97992 PMC5919879 29509546

[201] Kandalaft LE Odunsi K Coukos G. Immune therapy opportunities in ovarian cancer. Am Soc Clin Oncol Educ Book. (2020) 40:1–13. doi: 10.1200/EDBK_280539 32412818

[202] Santoiemma PP Reyes C Wang LP McLane MW Feldman MD Tanyi JL. Systematic evaluation of multiple immune markers reveals prognostic factors in ovarian cancer. Gynecol Oncol. (2016) 143:120–7. doi: 10.1016/j.ygyno.2016.07.105 27470997

[203] Garg V Oza AM. Treatment of ovarian cancer beyond PARP inhibition: current and future options. Drugs. (2023) 83:1365–85. doi: 10.1007/s40265-023-01934-0 PMC10581945 37737434

[204] Yang C Xia BR Zhang ZC Zhang YJ Lou G Jin WL. Immunotherapy for ovarian cancer: adjuvant, combination, and neoadjuvant. Front Immunol. (2020) 11:577869. doi: 10.3389/fimmu.2020.577869 33123161 PMC7572849

[205] Monk BJ Colombo N Oza AM Fujiwara K Birrer MJ Randall L. Chemotherapy with or without avelumab followed by avelumab maintenance versus chemotherapy alone in patients with previously untreated epithelial ovarian cancer (JAVELIN Ovarian 100): an open-label, randomised, phase 3 trial. Lancet Oncol. (2021) 22:1275–89. doi: 10.1016/S1470-2045(21)00342-9 34363762

[206] Pujade-Lauraine E Fujiwara K Ledermann JA Oza AM Kristeleit R Ray-Coquard IL. Avelumab alone or in combination with chemotherapy versus chemotherapy alone in platinum-resistant or platinum-refractory ovarian cancer (JAVELIN Ovarian 200): an open-label, three-arm, randomised, phase 3 study. Lancet Oncol. (2021) 22:1034–46. doi: 10.1016/S1470-2045(21)00216-3 34143970

[207] Moore KN Bookman M Sehouli J Miller A Anderson C Scambia G. Atezolizumab, bevacizumab, and chemotherapy for newly diagnosed stage III or IV ovarian cancer: placebo-controlled randomized phase III trial (IMagyn050/GOG 3015/ENGOT-OV39). J Clin Oncol. (2021) 39:1842–55. doi: 10.1200/JCO.21.00306 PMC8189598 33891472

[208] Kurtz JE Pujade-Lauraine E Oaknin A Belin L Leitner K Cibula D. Atezolizumab combined with bevacizumab and platinum-based therapy for platinum-sensitive ovarian cancer: placebo-controlled randomized phase III ATALANTE/ENGOT-ov29 trial. J Clin Oncol. (2023) 41:4768–78. doi: 10.1200/JCO.23.00529 PMC10602539 37643382

[209] Domchek SM Postel-Vinay S Im SA Park YH Delord JP Italiano A. Olaparib and durvalumab in patients with germline BRCA-mutated metastatic breast cancer (MEDIOLA): an open-label, multicentre, phase 1/2, basket study. Lancet Oncol. (2020) 21:1155–64. doi: 10.1016/S1470-2045(20)30324-7 32771088

[210] Konstantinopoulos PA Waggoner S Vidal GA Mita M Moroney JW Holloway R. Single-arm phases 1 and 2 trial of niraparib in combination with pembrolizumab in patients with recurrent platinum-resistant ovarian carcinoma. JAMA Oncol. (2019) 5:1141–9. doi: 10.1001/jamaoncol.2019.1048 PMC6567832 31194228

[211] Randall LM O'Malley DM Monk BJ Coleman RL Gaillard S Adams SF. MOONSTONE/GOG-3032: Interim analysis of a phase 2 study of niraparib + dostarlimab in patients (pts) with platinum-resistant ovarian cancer (PROC). J Clin Oncol. (2022) 40:5573. doi: 10.1200/JCO.2022.40.16_suppl.5573

[212] Lampert EJ Zimmer A Padget M Cimino-Mathews A Nair JR Liu Y. Combination of PARP inhibitor olaparib, and PD-L1 inhibitor durvalumab, in recurrent ovarian cancer: a proof-of-concept phase II study. Clin Cancer Res. (2020) 26:4268–79. doi: 10.1158/1078-0432.CCR-20-0056 PMC7442720 32398324

[213] Harter P Trillsch F Okamoto A Reuss A Kim J-W Rubio-Pérez MJ. Durvalumab with paclitaxel/carboplatin (PC) and bevacizumab (bev), followed by maintenance durvalumab, bev, and olaparib in patients (pts) with newly diagnosed advanced ovarian cancer (AOC) without a tumor BRCA1/2 mutation (non-tBRCAm): Results from the randomized, placebo (pbo)-controlled phase III DUO-O trial. J Clin Oncol. (2023) 41:LBA5506–LBA. doi: 10.1200/JCO.2023.41.17_suppl.LBA5506

[214] Vaishampayan UN Tomczak P Muzaffar J Winer IS Rosen SD Hoimes CJ. Nemvaleukin alfa monotherapy and in combination with pembrolizumab in patients (pts) with advanced solid tumors: ARTISTRY-1. J Clin Oncol. (2022) 40:2500. doi: 10.1200/JCO.2022.40.16_suppl.2500

[215] Moore KN Bouberhan S Hamilton EP Liu JF O'Cearbhaill RE O'Malley DM. First-in-human phase 1/2 study of ubamatamab, a MUC16xCD3 bispecific antibody, administered alone or in combination with cemiplimab in patients with recurrent ovarian cancer. J Clin Oncol. (2023) 41:TPS5624–TPS. doi: 10.1200/JCO.2023.41.16_suppl.TPS5624

[216] Veneziani A Lheureux S Alqaisi H Bhat G Colombo I Gonzalez E. Pembrolizumab, maveropepimut-S, and low-dose cyclophosphamide in advanced epithelial ovarian cancer: Results from phase 1 and expansion cohort of PESCO trial. J Clin Oncol. (2022) 40:5505. doi: 10.1200/JCO.2022.40.16_suppl.5505

[217] Miliotou AN Papadopoulou LC. CAR T-cell therapy: A new era in cancer immunotherapy. Curr Pharm Biotechnol. (2018) 19:5–18. doi: 10.2174/1389201019666180418095526 29667553

[218] Zhang XW Wu YS Xu TM Cui MH. CAR-T cells in the treatment of ovarian cancer: A promising cell therapy. Biomolecules. (2023) 13:465. doi: 10.3390/biom13030465 36979400 PMC10046142

[219] Kandalaft LE Powell DJ Jr. Coukos G. A phase I clinical trial of adoptive transfer of folate receptor-alpha redirected autologous T cells for recurrent ovarian cancer. J Transl Med. (2012) 10:157. doi: 10.1186/1479-5876-10-157 22863016 PMC3439340

[220] Hartkopf AD Fehm T Wallwiener M Lauer U. Oncolytic viruses to treat ovarian cancer patients - a review of results from clinical trials. Geburtshilfe Frauenheilkd. (2012) 72:132–6. doi: 10.1055/s-0031-1298281 PMC4215573 25374430

[221] Hoare J Campbell N Carapuca E. Oncolytic virus immunotherapies in ovarian cancer: moving beyond adenoviruses. Porto BioMed J. (2018) 3:e7. doi: 10.1016/j.pbj.0000000000000007 31595233 PMC6726300

[222] Gonzalez-Pastor R Goedegebuure PS Curiel DT. Understanding and addressing barriers to successful adenovirus-based virotherapy for ovarian cancer. Cancer Gene Ther. (2021) 28:375–89. doi: 10.1038/s41417-020-00227-y PMC8119242 32951021

[223] Moore KN Oza AM Colombo N Oaknin A Scambia G Lorusso D. randomized trial of mirvetuximab soravtansine versus chemotherapy in patients with platinum-resistant ovarian cancer: primary analysis of FORWARD I. Ann Oncol. (2021) 32:757–65. doi: 10.1016/j.annonc.2021.02.017 33667670

[224] Richardson DL Moore KN Vergote I Gilbert L Martin LP Mantia-Smaldone GM. Phase 1b study of mirvetuximab soravtansine, a folate receptor alpha (FRalpha)-targeting antibody-drug conjugate, in combination with carboplatin and bevacizumab in patients with platinum-sensitive ovarian cancer. Gynecol Oncol. (2024) 185:186–93. doi: 10.1016/j.ygyno.2024.01.045 38447347

[225] Matulonis UA Lorusso D Oaknin A Pignata S Dean A Denys H. Efficacy and safety of mirvetuximab soravtansine in patients with platinum-resistant ovarian cancer with high folate receptor alpha expression: results from the SORAYA study. J Clin Oncol. (2023) 41:2436–45. doi: 10.1200/JCO.22.01900 PMC10150846 36716407

[226] Banerjee S Oza AM Birrer MJ Hamilton EP Hasan J Leary A. Anti-NaPi2b antibody-drug conjugate lifastuzumab vedotin (DNIB0600A) compared with pegylated liposomal doxorubicin in patients with platinum-resistant ovarian cancer in a randomized, open-label, phase II study. Ann Oncol. (2018) 29:917–23. doi: 10.1093/annonc/mdy023 29401246

[227] Richardson D Hamilton E Barve M Anderson C Taylor S Lakhani N. Updated results from the phase 1 expansion study of upifitamab rilsodotin (UpRi; XMT-1536), a naPi2b-directed dolaflexin antibody drug conjugate (ADC) in ovarian cancer (076). Gynecologic Oncol. (2022) 166:S48. doi: 10.1016/S0090-8258(22)01294-X

[228] de Bono JS Concin N Hong DS Thistlethwaite FC Machiels JP Arkenau HT. Tisotumab vedotin in patients with advanced or metastatic solid tumours (InnovaTV 201): a first-in-human, multicentre, phase 1-2 trial. Lancet Oncol. (2019) 20:383–93. doi: 10.1016/S1470-2045(18)30859-3 30745090

[229] Lheureux S Alqaisi H Cohn DE Chern J-Y Duska LR Jewell A. A randomized phase II study of bevacizumab and weekly anetumab ravtansine or weekly paclitaxel in platinum-resistant or refractory ovarian cancer NCI trial10150. J Clin Oncol. (2022) 40:5514. doi: 10.1200/JCO.2022.40.16_suppl.5514

[230] Conlon NT Möhrle E Stockmann L Crown J. Abstract B095: Sacituzumab govitecan-based drug combinations overcome platinum/PARP inhibitor resistance in ovarian cancer models. Mol Cancer Ther. (2023) 22:B095–B. doi: 10.1158/1535-7163.TARG-23-B095

[231] Yap TA Hamilton E Bauer T Dumbrava EE Jeselsohn R Enke A. Phase ib SEASTAR study: combining rucaparib and sacituzumab govitecan in patients with cancer with or without mutations in homologous recombination repair genes. JCO Precis Oncol. (2022) 6:e2100456. doi: 10.1200/PO.21.00456 35138920 PMC8865521

[232] Munster PN Greenstein AE Fleming GF Borazanci E Sharma MR Custodio JM. Overcoming taxane resistance: preclinical and phase 1 studies of relacorilant, a selective glucocorticoid receptor modulator, with nab-paclitaxel in solid tumors. Clin Cancer Res. (2022) 28:3214–24. doi: 10.1158/1078-0432.CCR-21-4363 PMC9662918 35583817

[233] Colombo N Nguyen DD Fleming GF Grisham RN Lorusso D Van Gorp T. 721O Relacorilant, a selective glucocorticoid receptor modulator, in combination with nab-paclitaxel improves progression-free survival in patients with recurrent platinum-resistant ovarian cancer: A 3-arm, randomized, open-label, phase II study. Ann Oncol. (2021) 32:S725. doi: 10.1016/j.annonc.2021.08.1164

[234] Colombo N Gorp TV Matulonis UA Oaknin A Grisham RN Fleming GF. Overall survival data from a 3-arm, randomized, open-label, phase 2 study of relacorilant, a selective glucocorticoid receptor modulator, combined with nab-paclitaxel in patients with recurrent platinum-resistant ovarian cancer. J Clin Oncol. (2022) 40:LBA5503–LBA. doi: 10.1200/JCO.2022.40.17_suppl.LBA5503

[235] Rankin EB Fuh KC Taylor TE Krieg AJ Musser M Yuan J. AXL is an essential factor and therapeutic target for metastatic ovarian cancer. Cancer Res. (2010) 70:7570–9. doi: 10.1158/0008-5472.CAN-10-1267 PMC3408227 20858715

[236] Fuh KC Bookman MA Liu JF Coleman RL Herzog TJ Thaker PH. Phase 1b study of AVB-500 in combination with paclitaxel or pegylated liposomal doxorubicin platinum-resistant recurrent ovarian cancer. Gynecol Oncol. (2021) 163:254–61. doi: 10.1016/j.ygyno.2021.08.020 34474927

[237] Moufarrij S O'Cearbhaill RE. Novel therapeutics in ovarian cancer: expanding the toolbox. Curr Oncol. (2023) 31:97–114. doi: 10.3390/curroncol31010007 38248092 PMC10814452

[238] Moubarak M Harter P Ataseven B Traut A Welz J Baert T. Re-treatment with PARPi in patients with recurrent epithelial ovarian cancer: A single institutional experience. Gynecol Oncol Rep. (2022) 40:100939. doi: 10.1016/j.gore.2022.100939 35169607 PMC8829558

[239] Essel KG Behbakht K Lai T Hand L Evans E Dvorak J. PARPi after PARPi in epithelial ovarian cancer. Gynecol Oncol Rep. (2021) 35:100699. doi: 10.1016/j.gore.2021.100699 33537389 PMC7840844

[240] Xiong Y Guo Y Liu Y Wang H Gong W Liu Y. Pamiparib is a potent and selective PARP inhibitor with unique potential for the treatment of brain tumor. Neoplasia. (2020) 22:431–40. doi: 10.1016/j.neo.2020.06.009 PMC7350150 32652442

[241] Cecere SC Musacchio L Bartoletti M Salutari V Arenare L Lorusso D. Cytoreductive surgery followed by chemotherapy and olaparib maintenance in BRCA 1/2 mutated recurrent ovarian cancer: a retrospective MITO group study. Int J Gynecol Cancer. (2021) 31:1031–6. doi: 10.1136/ijgc-2020-002343 33990353

[242] Marchetti C Rosati A Scaletta G Pietragalla A Arcieri M Ergasti R. Secondary cytoreductive surgery in platinum-sensitive recurrent ovarian cancer before olaparib maintenance: Still getting any benefit? A case-control study. Gynecol Oncol. (2019) 155:400–5. doi: 10.1016/j.ygyno.2019.09.020 31606285

[243] Schettino C Musacchio L Bartoletti M Chiodini P Arenare L Baldassarre G. Olaparib beyond progression compared with platinum chemotherapy after secondary cytoreductive surgery in patients with recurrent ovarian cancer: phase III randomized, open-label MITO 35b study, a project of the MITO-MANGO groups. Int J Gynecol Cancer. (2022) 32:799–803. doi: 10.1136/ijgc-2022-003435 35318277

[244] Liu JF Barry WT Wenham RM Hendrickson AEW Armstrong DK Chan N. A phase 2 biomarker trial of combination cediranib and olaparib in relapsed platinum (plat) sensitive and plat resistant ovarian cancer (ovca). J Clin Oncol. (2018) 36:5519. doi: 10.1200/JCO.2018.36.15_suppl.5519

[245] Dellavedova G Decio A Formenti L Albertella MR Wilson J Staniszewska AD. The PARP1 inhibitor AZD5305 impairs ovarian adenocarcinoma progression and visceral metastases in patient-derived xenografts alone and in combination with carboplatin. Cancer Res Commun. (2023) 3:489–500. doi: 10.1158/2767-9764.CRC-22-0423 36994441 PMC10042207

[246] Illuzzi G Staniszewska AD Gill SJ Pike A McWilliams L Critchlow SE. Preclinical characterization of AZD5305, A next-generation, highly selective PARP1 inhibitor and trapper. Clin Cancer Res. (2022) 28:4724–36. doi: 10.1158/1078-0432.CCR-22-0301 PMC9623235 35929986

[247] Yap TA Im S-A Schram AM Sharp A Balmana J Baird RD. Abstract CT007: PETRA: First in class, first in human trial of the next generation PARP1-selective inhibitor AZD5305 in patients (pts) with BRCA1/2, PALB2 or RAD51C/D mutations. Cancer Res. (2022) 82:CT007–CT. doi: 10.1158/1538-7445.AM2022-CT007

[248] Ahmed AA Etemadmoghadam D Temple J Lynch AG Riad M Sharma R. Driver mutations in TP53 are ubiquitous in high grade serous carcinoma of the ovary. J Pathol. (2010) 221:49–56. doi: 10.1002/path.2696 20229506 PMC3262968

[249] Mohni KN Thompson PS Luzwick JW Glick GG Pendleton CS Lehmann BD. A synthetic lethal screen identifies DNA repair pathways that sensitize cancer cells to combined ATR inhibition and cisplatin treatments. PloS One. (2015) 10:e0125482. doi: 10.1371/journal.pone.0125482 25965342 PMC4428765

[250] Biegala L Gajek A Szymczak-Pajor I Marczak A Sliwinska A Rogalska A. Targeted inhibition of the ATR/CHK1 pathway overcomes resistance to olaparib and dysregulates DNA damage response protein expression in BRCA2(MUT) ovarian cancer cells. Sci Rep. (2023) 13:22659. doi: 10.1038/s41598-023-50151-y 38114660 PMC10730696

[251] Li S Wang T Fei X Zhang M. ATR inhibitors in platinum-resistant ovarian cancer. Cancers (Basel). (2022) 14:5902. doi: 10.3390/cancers14235902 36497387 PMC9740197

[252] Konstantinopoulos PA Cheng SC Wahner Hendrickson AE Penson RT Schumer ST Doyle LA. Berzosertib plus gemcitabine versus gemcitabine alone in platinum-resistant high-grade serous ovarian cancer: a multicentre, open-label, randomised, phase 2 trial. Lancet Oncol. (2020) 21:957–68. doi: 10.1016/S1470-2045(20)30180-7 PMC8023719 32553118

[253] Wethington SL Shah PD Martin L Tanyi JL Latif N Morgan M. (ceralasertib) and PARP (olaparib) inhibitor (CAPRI) trial in acquired PARP inhibitor-resistant homologous recombination-deficient ovarian cancer. Clin Cancer Res. (2023) 29:2800–7. doi: 10.1158/1078-0432.CCR-22-2444 PMC11934101 37097611

[254] Lloyd RL Urban V Munoz-Martinez F Ayestaran I Thomas JC de Renty C. Loss of Cyclin C or CDK8 provides ATR inhibitor resistance by suppressing transcription-associated replication stress. Nucleic Acids Res. (2021) 49:8665–83. doi: 10.1093/nar/gkab628 PMC8421211 34329458

[255] Guzi TJ Paruch K Dwyer MP Labroli M Shanahan F Davis N. Targeting the replication checkpoint using SCH 900776, a potent and functionally selective CHK1 inhibitor identified via high content screening. Mol Cancer Ther. (2011) 10:591–602. doi: 10.1158/1535-7163.MCT-10-0928 21321066

[256] Otto T Sicinski P. Cell cycle proteins as promising targets in cancer therapy. Nat Rev Cancer. (2017) 17:93–115. doi: 10.1038/nrc.2016.138 28127048 PMC5345933

[257] Lee JM Nair J Zimmer A Lipkowitz S Annunziata CM Merino MJ. Prexasertib, a cell cycle checkpoint kinase 1 and 2 inhibitor, in BRCA wild-type recurrent high-grade serous ovarian cancer: a first-in-class proof-of-concept phase 2 study. Lancet Oncol. (2018) 19:207–15. doi: 10.1016/S1470-2045(18)30009-3 PMC7366122 29361470

[258] Konstantinopoulos PA Lee JM Gao B Miller R Lee JY Colombo N. A Phase 2 study of prexasertib (LY2606368) in platinum resistant or refractory recurrent ovarian cancer. Gynecol Oncol. (2022) 167:213–25. doi: 10.1016/j.ygyno.2022.09.019 PMC10673677 36192237

[259] Zhao Y Zhou K Xia X Guo Y Tao L. Chk1 inhibition-induced BRCAness synergizes with olaparib in p53-deficient cancer cells. Cell Cycle. (2022) 22:200–12. doi: 10.1080/15384101.2022.2111769 PMC9815235 35959961

[260] Matheson CJ Backos DS Reigan P. Targeting WEE1 kinase in cancer. Trends Pharmacol Sci. (2016) 37:872–81. doi: 10.1016/j.tips.2016.06.006 27427153

[261] Moore KN Chambers SK Hamilton EP Chen L-m Oza AM Ghamande SA. Adavosertib with chemotherapy (CT) in patients (pts) with platinum-resistant ovarian cancer (PPROC): An open label, four-arm, phase II study. J Clin Oncol. (2019) 37:5513. doi: 10.1200/JCO.2019.37.15_suppl.5513 34645648

[262] Moore KN Chambers SK Hamilton EP Chen LM Oza AM Ghamande SA. Adavosertib with chemotherapy in patients with primary platinum-resistant ovarian, fallopian tube, or peritoneal cancer: an open-label, four-arm, phase II study. Clin Cancer Res. (2022) 28:36–44. doi: 10.1158/1078-0432.CCR-21-0158 34645648

[263] Oza AM Estevez-Diz M Grischke EM Hall M Marme F Provencher D. A biomarker-enriched, randomized phase II trial of adavosertib (AZD1775) plus paclitaxel and carboplatin for women with platinum-sensitive TP53-mutant ovarian cancer. Clin Cancer Res. (2020) 26:4767–76. doi: 10.1158/1078-0432.CCR-20-0219 32611648

[264] Lheureux S Cristea MC Bruce JP Garg S Cabanero M Mantia-Smaldone G. Adavosertib plus gemcitabine for platinum-resistant or platinum-refractory recurrent ovarian cancer: a double-blind, randomised, placebo-controlled, phase 2 trial. Lancet. (2021) 397:281–92. doi: 10.1016/S0140-6736(20)32554-X PMC10792546 33485453

[265] Au-Yeung G Bressel M Prall O Opar P Andrews J Mongta S. PO003/#269 Ignite: a phase II signal-seeking trial of adavosertib targeting recurrent high grade serous ovarian cancer with cyclin E1 over-expression with and without gene amplification. Int J Gynecol Cancer. (2023) 33(Suppl 4):A2–3. doi: 10.1136/ijgc-2023-IGCS.3

[266] Westin SN Coleman RL Fellman BM Yuan Y Sood AK Soliman PT. EFFORT: EFFicacy Of adavosertib in parp ResisTance: A randomized two-arm non-comparative phase II study of adavosertib with or without olaparib in women with PARP-resistant ovarian cancer. J Clin Oncol. (2021) 39:5505. doi: 10.1200/JCO.2021.39.15_suppl.5505

[267] Zhang C Peng K Liu Q Huang Q Liu T. Adavosertib and beyond: Biomarkers, drug combination and toxicity of WEE1 inhibitors. Crit Rev Oncol Hematol. (2024) 193:104233. doi: 10.1016/j.critrevonc.2023.104233 38103761

[268] Gallo D Young JTF Fourtounis J Martino G Alvarez-Quilon A Bernier C. CCNE1 amplification is synthetic lethal with PKMYT1 kinase inhibition. Nature. (2022) 604:749–56. doi: 10.1038/s41586-022-04638-9 PMC9046089 35444283

[269] Kang EY Weir A Meagher NS Farrington K Nelson GS Ghatage P. CCNE1 and survival of patients with tubo-ovarian high-grade serous carcinoma: An Ovarian Tumor Tissue Analysis consortium study. Cancer. (2023) 129:697–713. doi: 10.1002/cncr.34582 36572991 PMC10107112

[270] Szychowski J Papp R Dietrich E Liu B Vallee F Leclaire ME. Discovery of an orally bioavailable and selective PKMYT1 inhibitor, RP-6306. J Med Chem. (2022) 65:10251–84. doi: 10.1021/acs.jmedchem.2c00552 PMC9837800 35880755

[271] Barszczewska-Pietraszek G Drzewiecka M Czarny P Skorski T Sliwinski T. Poltheta inhibition: an anticancer therapy for HR-deficient tumours. Int J Mol Sci. (2022) 24:319. doi: 10.3390/ijms24010319 36613762 PMC9820168

[272] Sun C Fang Y Yin J Chen J Ju Z Zhang D. Rational combination therapy with PARP and MEK inhibitors capitalizes on therapeutic liabilities in RAS mutant cancers. Sci Transl Med. (2017) 9:eaal5148. doi: 10.1126/scitranslmed.aal5148 28566428 PMC5919217

[273] Vena F Jia R Esfandiari A Garcia-Gomez JJ Rodriguez-Justo M Ma J. MEK inhibition leads to BRCA2 downregulation and sensitization to DNA damaging agents in pancreas and ovarian cancer models. Oncotarget. (2018) 9:11592–603. doi: 10.18632/oncotarget.v9i14 PMC5837749 29545922

[274] Yang B Li X Fu Y Guo E Ye Y Li F. MEK inhibition remodels the immune landscape of mutant KRAS tumors to overcome resistance to PARP and immune checkpoint inhibitors. Cancer Res. (2021) 81:2714–29. doi: 10.1158/0008-5472.CAN-20-2370 PMC8265237 33589518

[275] Kosiol N Juranek S Brossart P Heine A Paeschke K. G-quadruplexes: a promising target for cancer therapy. Mol Cancer. (2021) 20:40. doi: 10.1186/s12943-021-01328-4 33632214 PMC7905668

[276] Xu H Di Antonio M McKinney S Mathew V Ho B O'Neil NJ. CX-5461 is a DNA G-quadruplex stabilizer with selective lethality in BRCA1/2 deficient tumours. Nat Commun. (2017) 8:14432. doi: 10.1038/ncomms14432 28211448 PMC5321743

[277] Bryan TM. Mechanisms of DNA replication and repair: insights from the study of G-quadruplexes. Molecules. (2019) 24:3439. doi: 10.3390/molecules24193439 31546714 PMC6804030

[278] Hilton J Gelmon K Bedard PL Tu D Xu H Tinker AV. Results of the phase I CCTG IND.231 trial of CX-5461 in patients with advanced solid tumors enriched for DNA-repair deficiencies. Nat Commun. (2022) 13:3607. doi: 10.1038/s41467-022-31199-2 35750695 PMC9232501

[279] Kogan AA McLaughlin LJ Topper M Muvarak N Stojanovic L Creed TM. DNA demethylating agents generate a brcaness effect in multiple sporadic tumor types: prediction for sensitivity to PARP inhibitors in AML. Blood. (2017) 130:3347. doi: 10.1182/blood.V130.Suppl_1.3347.3347

[280] Oza AM Matulonis UA Alvarez Secord A Nemunaitis J Roman LD Blagden SP. A randomized phase II trial of epigenetic priming with guadecitabine and carboplatin in platinum-resistant, recurrent ovarian cancer. Clin Cancer Res. (2020) 26:1009–16. doi: 10.1158/1078-0432.CCR-19-1638 PMC7056559 31831561

[281] Dizon DS Damstrup L Finkler NJ Lassen U Celano P Glasspool R. Phase II activity of belinostat (PXD-101), carboplatin, and paclitaxel in women with previously treated ovarian cancer. Int J Gynecol Cancer. (2012) 22:979–86. doi: 10.1097/IGC.0b013e31825736fd 22694911

[282] Doroshow DB Eder JP LoRusso PM. BET inhibitors: a novel epigenetic approach. Ann Oncol. (2017) 28:1776–87. doi: 10.1093/annonc/mdx157 28838216

[283] Karakashev S Zhu H Yokoyama Y Zhao B Fatkhutdinov N Kossenkov AV. BET bromodomain inhibition synergizes with PARP inhibitor in epithelial ovarian cancer. Cell Rep. (2017) 21:3398–405. doi: 10.1016/j.celrep.2017.11.095 PMC5745042 29262321

[284] Wilson AJ Stubbs M Liu P Ruggeri B Khabele D. The BET inhibitor INCB054329 reduces homologous recombination efficiency and augments PARP inhibitor activity in ovarian cancer. Gynecol Oncol. (2018) 149:575–84. doi: 10.1016/j.ygyno.2018.03.049 PMC5986599 29567272

